# Single-cell RNA sequencing reveals S100a9^hi^ macrophages promote the transition from acute inflammation to fibrotic remodeling after myocardial ischemia‒reperfusion

**DOI:** 10.7150/thno.91180

**Published:** 2024-01-20

**Authors:** Shichun Shen, Meng Zhang, Xiaohe Wang, Qiaoling Liu, Huimin Su, Bingyi Sun, Zhiqing Guo, Beiduo Tian, Hong Gan, Chen Gong, Likun Ma

**Affiliations:** 1 Department of Cardiology, The First Affiliated Hospital of USTC, Division of Life Sciences and Medicine, University of Science and Technology of China, Hefei, China.; 2 Department of Pediatrics, The First Affiliated Hospital of Anhui Medical University, Hefei, China.; 3 Department of Cardiology, The Second Affiliated Hospital of Anhui Medical University, Hefei, China.; 4 School of Cardiovascular and Metabolic Health, University of Glasgow, Glasgow, United Kingdom.; 5 The First Clinical Medical school of Anhui Medical university, Hefei, China.; 6 Department of Maternal, Child and Adolescent Health, School of Public Health, Anhui Medical University, No 81 Meishan Road, Hefei 230032, Anhui, China.; 7 MOE Key Laboratory of Population Health Across Life Cycle, Hefei, China.

**Keywords:** myocardial ischemia-reperfusion injury, inflammation, cardiac fibrosis, S100a9^hi^ macrophages, macrophage-to-myofibroblast transition

## Abstract

**Rationale:** The transition from acute inflammation to fibrosis following myocardial ischemia‒reperfusion (MIR) significantly affects prognosis. Macrophages play a pivotal role in inflammatory damage and repair after MIR. However, the heterogeneity and transformation mechanisms of macrophages during this transition are not well understood.

**Methods:** In this study, we used single-cell RNA sequencing (scRNA-seq) and mass cytometry to examine murine monocyte-derived macrophages after MIR to investigate macrophage subtypes and their roles in the MIR process. S100a9^-/-^ mice were used to establish MIR model to clarify the mechanism of alleviating inflammation and fibrosis after MIR. Reinfusion of bone marrow-derived macrophages (BMDMs) after macrophage depletion (MD) in mice subjected to MIR were performed to further examine the role of S100a9^hi^ macrophages in MIR.

**Results:** We identified a unique subtype of S100a9^hi^ macrophages that originate from monocytes and are involved in acute inflammation and fibrosis. These S100a9^hi^ macrophages infiltrate the heart as early as 2 h post-reperfusion and activate the Myd88/NFκB/NLRP3 signaling pathway, amplifying inflammatory responses. As the tissue environment shifts from proinflammatory to reparative, S100a9 activates transforming growth factor-β (Tgf-β)/p-smad3 signaling. This activation not only induces the transformation of myocardial fibroblasts to myofibroblasts but also promotes fibrosis via the macrophage-to-myofibroblast transition (MMT). Targeting S100a9 with a specific inhibitor could effectively mitigate acute inflammatory damage and halt the progression of fibrosis, including MMT.

**Conclusion:** S100a9^hi^ macrophages are a promising therapeutic target for managing the transition from inflammation to fibrosis after MIR.

## Introduction

Acute myocardial infarction (MI) is one of the leading causes of mortality worldwide 1. The development of coronary interventions and thrombolytic treatments has proven effective in limiting infarct size and enhancing clinical outcomes. However, the reperfusion of occluded vessels not only fails to restore myocardial damage caused by acute ischemic hypoxia but can also further exacerbate structural, functional, and electrophysiological impairments in the heart. These complications that arise from reperfusion therapy are termed myocardial ischemia‒reperfusion (MIR) injury 2. MIR injury involves the multifaceted interplay of inflammation, oxidative stress, and metabolic factors, and the damage can extend to up to 50% of the infarcted area 3.

Increasing evidence, including findings from our study, underscores the pivotal and indispensable roles of innate and adaptive immunity in acute MIR injury 4, 5. In the early stages, damaged myocardial tissues release a myriad of damage signals, activating innate immunity and promoting the infiltration and adhesion of circulating immune cells 6, 7. After infiltrating the injured tissue, Ly6c^hi^ monocytes differentiate into macrophages. These macrophages can express many pattern recognition receptors, such as Toll-like receptors, NOD-like receptors, and C-type lectin receptors 8. The activation of these receptors triggers the release of a plethora of inflammatory mediators and cytokines, leading to sterile inflammation and tissue injury.

Macrophages play an irreplaceable role in MIR injury. In the early phase of MIR, blood monocytes infiltrate damaged cardiac tissue and transform into macrophages. Initially, these monocyte-derived macrophages predominantly exhibit a proinflammatory M1 phenotype, releasing cytokines and inflammatory mediators that drive the early inflammatory phase 9, 10. Depending on disease progression, macrophages can exhibit different phenotypic characteristics. By the day 3 after MIR, reparative M2 macrophages dominate, suppressing the M1 macrophage-mediated inflammatory response 11. The exact mechanism underlying this phenotypic switch remains unclear, but our research, along with prior evidence, suggests that macrophage-derived transforming growth factor-β (Tgf-β) can mediate macrophage phenotypic transformation 12. During the reparative phase, M2 macrophages release high levels of Tgf-β, stimulating fibroblasts to transform into myofibroblasts, thereby promoting ventricular remodeling 13, 14. Fibrosis in the myocardium is a physiological repair mechanism that maintains the structural and functional integrity of the heart following a myocardial infarction. Proper fibrosis is essential for effectively preserving the integrity of the heart structure and preventing cardiac rupture. However, excessive fibrosis can lead to impaired cardiac contractile function, ultimately resulting in heart failure 15. While fibroblasts have traditionally been considered the primary effector cells in fibrotic remodeling, recent studies have identified macrophages that can transition into myofibroblasts, facilitating fibrosis in the retina 16, kidneys 17, pancreas 18, and tumors 19. This novel cellular phenotypic transition has also been observed in the heart 20, although the precise mechanism remains to be determine. Our findings shed light on the role of macrophage-to-myofibroblast transition (MMT) in fibrotic changes following MIR injury.

In our study, single-cell RNA sequencing (scRNA-seq) identified a subtype of monocyte-derived macrophages that extensively infiltrated damaged myocardial tissue after acute MIR. These macrophages highly express S100a9. In the early stages after MIR, these cells can activate the Myd88/NFκB/NLRP3 inflammatory signaling pathway, releasing a vast array of inflammatory mediators and cytokines, thereby amplifying the inflammatory response. Concurrently, S100a9^hi^ macrophages can activate fibroblasts to form myofibroblasts and undergo MMT through the Tgf-β/p-smad3 signaling pathway, thereby accelerating myocardial fibrosis. We further explored the impact of inhibiting S100a9 on acute injury and chronic fibrosis after MIR in mice using S100a9^-/-^ mice and S100a9 inhibitors.

## Experimental Section

### Animal Experiment

Male C57BL/6 mice (6-8 weeks, 20-24 g) were procured from the Animal Experiment Center of Anhui Medical University and housed in SPF conditions. All animal experiment protocols were approved by the Animal Ethics Committee of the First Affiliated Hospital of Anhui Medical University (Approval No: LLSC20210347). S100a9^-/-^ mouse purchased from the Division of Life Sciences and Medicine, University of Science and Technology of China were also employed in this research.

### Establishment of Mouse MIR Model

The mouse MIR model was established as per previous studies 21. Briefly, anesthesia was administered through isoflurane inhalation and mechanical ventilation. The heart was exposed between the 3^rd^ and 4^th^ ribs of the left sternum, and the left coronary artery was ligated for 30 min followed by reperfusion. The Sham group underwent the same surgical procedures except for the coronary ligation.

### Peripheral blood mononuclear cell (PBMC) Isolation

Fresh blood was collected from the left ventricle of the mice, centrifuged to remove plasma, and PBMC was isolated using Ficoll lymphocyte separation medium.

### Preparation of Cardiac Single-Cell Suspension

Left ventricular tissues of mice hearts including the infarct area and the ischemic risk area were collected at different time points post-MIR, as this region is significantly affected. The tissues were dissociated into single-cell suspensions using a mouse heart tissue dissociation kit (Miltenyi Biotec, Germany) as per the manufacturer's instructions. Post-dissociation, the suspensions were filtered through a 40 µm filter and subjected to red blood cell lysis (Solarbio, R1010).

### Flow Cytometry and scRNA-Seq

Single-cell suspensions from mouse heart tissues were incubated with CD45 (Biolegend, #157603) and CD11b (Biolegend, #101212) at 4 °C for 30 min, and mouse PBMCs were incubated with CD45 (Biolegend, #157603), CD11b (Biolegend, #101212), and Ly6c (Biolegend, #128017) at 4 °C for 30 min. Cells were then sorted using a flow cytometer (Beckman) to isolate CD45^+^CD11b^+^ and CD45^+^Ly6C^+^ cells for scRNA-Seq.

### scRNA-Seq by10 × Genomics

#### Single Cell Library Preparation and Sequencing

Single cell 3'gene expression profiling was performed by using Chromium Next GEM Single Cell 3ʹ Kit v3.1 (10x Genomics, #1000268) and Chromium Next GEM Chip G Single Cell Kit (10x Genomics, #1000120). Purified Cd45^+^Ly6c^+^/Cd11b^+^ cells were loaded onto the Chromium single cell controller (10x Genomics) to generate droplets encapsulating single cell and barcoded beads in the emulsion according to the manufacturer's protocol. Captured cells were lysed and the cDNA purified by Dynabeads (10X Genomics) was followed by PCR amplification. Cell-barcoded 3'gene expression libraries were sequenced on an Illumina NovaSeq6000 system (Illumina, San Diego, CA, USA) by Shanghai Applied Protein Technology Co., Ltd.

#### scRNA-Seq Data Processing

FASTQ data of individual samples were checked using FastQC v0.11.9. The Cell Ranger 'count' pipeline v7.0.0 was used to generate expression matrices from raw scRNA-seq data. Expression matrices from different batches were analyzed, integrated, and processed using Seurat v4.0.6. Quality control was performed to filter out barcodes with less than 200 genes and UMI counts exceeding 10% of the maximum value, and cells with over 30% mitochondrial genes. The UMI counts were log-normalized using the Seurat package v4.0.6 and the NormalizeData function. The FindVariableFeatures identified 2000 highly variable genes, which were normalized and scaled using the ScaleData function. Principal component analysis (PCA) was performed on these genes using the RunPCA function in Seurat. The Harmony algorithm v0.1.1 was used to integrate cells from different samples to mitigate batch effects. Post-integration, dimensionality reduction was performed using RunUMAP/RunTSNE functions on the top 30 principal components. Clustering was performed using FindNeighbors and FindClusters with a resolution of 0.8. The clusters obtained were initially annotated using the singleR software v1.8.0, and the results were further refined using canonical markers.

### Enrichment Analysis

The FindAllMarkers function with default settings was used to identify DEGs for each cluster. DEGs were categorized as upregulated or downregulated based on their average log2FC values being greater or less than 0.25, respectively. GO and KEGG enrichment analyses were performed on upregulated and downregulated DEGs using clusterProfiler v4.2.2.

### Pseudotime Inference using Monocle2

Monocle2 v1.0.0 was used to predict the trajectory of baseline cell types. Cell count expressions and features were extracted from the Seurat object to construct cell datasets. The DifferentialGeneTest function was used to filter genes that can define variation across cell groups. “Ordering genes” were selected by setting the fullModelStr to “~sm.ns”. After reducing dimensions using the reduceDimension function with DDRTree, cell trajectories were constructed using the orderCells function on variable genes.

### Scoring of Biological Processes

Based on previously reported datasets of functional features of monocytes/macrophages from different organ sources, individual cells were scored for functional features. This biological scoring was defined as the average normalized expression of gene features representing different biological functions. Gene functional datasets were collected from the Gene Ontology database, and differential genes were identified using the Wilcoxon test with an adjusted p-value cutoff of 0.05.

### Mass Cytometry

Single-cell suspensions were stained on ice using 0.25 µM Cell-ID™ Cisplatin-194Pt (Fluidigm, #201194) to discriminate between live and dead cells. Subsequently, cells were blocked with blocking solution on ice for 20 min to reduce non-specific binding. Surface antibody mix and 100 µl of intracellular antibody combination were sequentially added, and cells were incubated on ice for 30 min each for surface staining. After washing thrice with FACS buffer, cells were resuspended using EQ™ Four Element Calibration Beads (Fluidigm, #201078). Mass cytometry (Fluidigm, Helios) was performed by Zhejiang Puluoting Health Technology Co., Ltd. (Hangzhou, China) for analysis, and CyTOF data were analyzed on Cytobank.

### Macrophage Depletion (MD) in Mice

Clodronate liposomes (LIPOSOMA, #CP-005-005, Netherlands) and control liposomes were administered intravenously to C57BL/6 mice (10 ml/kg) 24 h prior to MIR.

### Measurement of Myocardial Infarct Area

The hearts of mice subjected to 2 h of reperfusion were ligated in situ, and 0.4% Evans blue (Sigma-Aldrich, USA) dye was injected via the internal jugular vein. Hearts were excised after 1 min, sliced, and immersed in 2% 2,3,5-Triphenyltetrazolium chloride (TTC, Sigma-Aldrich, USA) solution, incubated at 37 °C for 10 min, and scanned for data recording.

### Myocardial Injury Marker Detection

Mouse Lactate Dehydrogenase (LDH) assay kit (Jiancheng, China), mouse Creatine Kinase Myocardial Band (CKMB) isoenzyme assay kits (Jiancheng, China), and mouse cardiac Troponin I (cTnI) ELISA kit (Elabscience, China) were used to assess the level of myocardial injury in mice.

### Cytokine and Chemokine Quantification

Bio-Plex Pro Mouse Chemokine Panel 31-Plex kit (Bio-Rad, #12009159, Hercules, CA, USA) was employed to quantify cytokine and chemokine concentrations in mouse left ventricular tissue homogenates at different time points post-MIR, following the manufacturer's instructions. The plates were read using the Luminex X200 system (Luminex Corporation, Austin, Texas, USA).

### Histopathology

To evaluate morphological and fibrotic changes in the heart tissue post-MIR, hearts from different groups of mice were collected, paraffin-embedded, sectioned, and stained with Hematoxylin-Eosin (H&E) and Masson's Trichrome.

### TUNEL Staining

Tissue sections were processed with xylene, ethanol, and then incubated with 20 µg/ml Proteinase K without DNase at 37 °C for 30 min. 50 µl of Tunel detection fluid was added and incubated at 37 °C for 60 min. DAPI was used for nuclear counterstaining, and slides were observed under a microscope.

### Drug Treatment

24 h prior to MIR, mice were intraperitoneally injected with 5 mg/kg/d tasquinimod (MCE, #ABR-215050) diluted in a carrier solution (10% DMSO, 40% PEG300, and 5% Tween-80 in PBS solution), and treated until 24 h before sacrifice.

### Echocardiography

After continuous gas anesthesia using an anesthesia machine and ventilator, mice were placed on a heating pad. Cardiac function was assessed and echocardiographic data were analyzed by using VINNO (VINNO, Suzhou, China).

### Bone marrow-derived macrophages (BMDMs) Extraction and Induction Culture

BMDMs were flushed from the tibiae and femurs of 6-8 weeks old C57BL/6 mice with PBS. The cell suspension was filtered through a 70 µm cell strainer and subjected to red blood cell lysis (Solarbio, #R1010). Cells were cultured in 1640 medium containing macrophage-stimulating factor (M-CSF, Peprotech, USA) for 7 days before subsequent experiments. M1 phenotype was induced with 1 µg/ml LPS (Sigma-Aldrich, USA), and M2 phenotype with Interleukin-4 (IL-4, Peprotech, USA). For exploring Tgf-β induced macrophage phenotype transition, cells were stimulated with 1 µg/ml LPS for 24 h, followed by 5 µg/ml Tgf-β (SinoBiological, China) for another 24 h. For MMT, mature induced BMDMs were treated with 5 µg/ml Tgf-β for 5 days before subsequent experiments.

### Primary cardiac fibroblasts (PCFs) extraction

Newborn C57BL/6 mouse aged 1-3 days were sacrificed and their hearts were collected. The heart was digested with 0.25% trypsin in 37 ℃ until the tissue was completely digested. After centrifuged at 1500 rpm for 5 min, cells were resuspended in DMEM medium containing 10% serum and cultured in 37 ℃ for 1 h. Discarding the medium and the adherent cells are PCFs. PCFs from passages 2-4 were used for subsequent experiments.

### Co-culture of BMDMs and PCFs

To investigate the effect of BMDMs on PCFs, BMDMs were seeded in the 0.4 μm polyester membrane Transwell inserts suitable for six-well plates, and the Transwell chamber containing BMDMs was inserted into the six-well plate for co-culturing with PCFs for 24 h.

### Adoptive Transfer Studies

To ascertain the role of S100a9^hi^ macrophages in myocardial fibrosis, BMDMs from wild-type (WT) and S100a9^-/-^ mice were intravenously infused into macrophage-depleted mice (1×10^6^ cells per mouse) on the day 4 post-MIR.

### Immunohistochemistry

Cardiac tissues were fixed in 4% paraformaldehyde, followed by paraffin embedding and sectioning into 5 µm slices. The sections underwent deparaffinization and antigen retrieval. Primary antibodies against Ly6g (Abcam, #ab238132, 1:1000) were utilized to measure the levels of neutrophil in murine cardiac tissue, respectively.

### Immunofluorescence

Following fixation in 4% paraformaldehyde, cardiac tissues were embedded in paraffin and sectioned into 5 µm slices. After deparaffinization and antigen retrieval, endogenous peroxidase activity was blocked using 3% hydrogen peroxide. According to the TSA cyclic staining method, sections were incubated sequentially with primary antibodies against CD68 (CST, #97778, 1:400), Ccl2 (Proteintech, #26161-1-AP, 1:200), S100a9 (Proteintech, #26992-1-AP, 1:400), Ly6g (Abcam, #ab238132, 1:400), Myd88 (CST, #4383S, 1:200), Arg-1 (CST, #93668S, 1:200), α-SMA (CST, #48938, 1:200), p-smad3 (Abcam, #ab52903, 1:200), iNos (CST, #13120, 1:400), and corresponding secondary antibodies, followed by incubation with fluorophore-conjugated TSA dye. Sections were counterstained with DAPI and observed under a fluorescence microscope.

PCFs cultures were prepared and fixed with paraformaldehyde for 15 min. Co-incubation with α-SMA (CST, #48938, 1:200) and Col1α1 (CST, #72026, 1:200) primary antibodies was performed overnight, followed by 1 h incubation with corresponding fluorophore-conjugated secondary antibodies. After sealing with a DAPI-containing mounting medium, observations were made under a fluorescence microscope.

### Western Blotting

Tissues or cells were lysed using RIPA lysis buffer, and protein concentrations were determined using a BCA Protein Assay Kit (Yamei, #ZJ101). Proteins were electrophoresed in SDS gels and transferred onto PVDF membranes. Membranes were incubated overnight at 4 °C with primary antibodies against S100a9 (Proteintech, #26992-1-AP, 1:1000), Phospho-NFκB (CST, #3033s, 1:1000), Myd88 (CST, #4283s, 1:1000), NLRP3 (ABCAM, #ab263899, 1:1000), Caspase-1 (Proteintech, #22915-1-AP, 1:1000), TNF-α (Proteintech, #60291-1-Ig, 1:1000), IL-1β (Proteintech, #16806-1-AP, 1:1000), p-smad3 (Abcam, #ab52903, 1:1000), t-smad3 (Proteintech, #66516-1-Ig, 1:1000), Tgf-β (Proteintech, #21898-1-AP, 1:1000), α-SMA (CST, #48938, 1:1000), Arg-1(CST, #93668S, 1:1000) β-actin (Proteintech, #66009-1-Ig, 1:1000), GAPDH (Proteintech, #10494-1-AP, 1:1000). Following incubation with corresponding secondary antibodies, detection was performed on a Biorad imaging system. Quantification of target protein expression levels was conducted using ImageJ, with analyses normalized to loading control expression.

### Statistical Analysis

Data following a normal distribution were presented as mean ± standard deviation (SD), while non-normally distributed data were presented as median and quartiles. Comparisons between groups with normally distributed data were performed using one-way ANOVA, with Bonferroni post-hoc tests for subgroup comparisons. Student's t test analyses were employed for comparisons between two groups. All statistical analyses and graphical representations were conducted using SPSS 16.0 and GraphPad Prism 9.0 (GraphPad Software, Inc., La Jolla, California, USA), respectively.

## Results

### The Mononuclear Phagocyte Atlas at Single-Cell Resolution Following MIR in Mice

**Figure 1:**
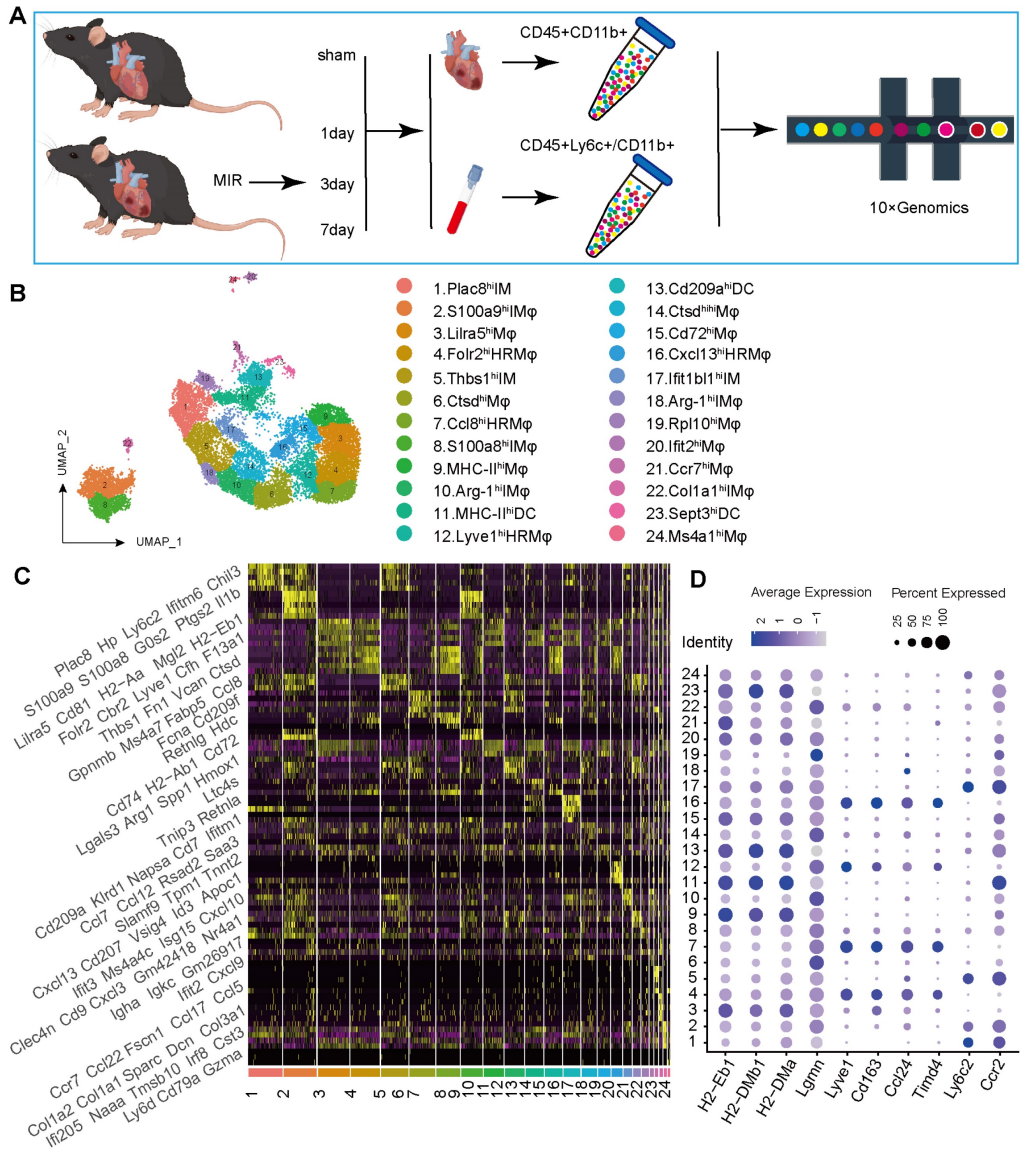
** ScRNA-seq of cardiac and Peripheral blood MPCs from six samples in the MIR data set and reveals the post-MIR dynamics of MPCs.** (A) The flow chart of experimental design. n = 9 mice at each time point. The flow chart was drawn by Figdraw. (B) UMAP plot of 17393 cells pooled from Sham and three MIR groups, showing the MPCs identified based on the canonical marker genes. (C) Heatmap of top five differentially expressed genes in each subpopulation. (D) Dot plot of monocyte, heart resident macrophage and monocyte-derived infiltrated macrophages markers in each MPC cluster.

To determine the potential sources of and dynamic changes in cardiac mononuclear phagocytic cells (MPCs) after MIR, we conducted a time-series scRNA-seq analysis of MPCs from mouse hearts and blood on days 1, 3 and 7 after MIR (**Figure** 1A). To maximize MPCs collection from the blood, we harvested Cd45^+^ly6c^+^ and Cd45^+^Cd11b^+^ cells in the blood, mixed them in a 1:1 ratio, and subjected them to scRNA-seq analysis. Due to the limited number of F4/80^+^ cells in the heart, we sorted Cd45^+^Cd11b^+^ cells in heart for scRNA-seq using 10X Genomics (**Figure** S1A-B).

The scRNA-seq results showed a total of 38,497 cells that passed quality control and were classified into 26 clusters (**Figure** S2A-B, **Table** S1). High expression of specific genes in these cells is shown in **Figure** S2C. Based on representative MPC genes, cells with high expression of CD68, Adgre1, Cx3cr1, Itgax, and Cd209a were defined as MPCs (**Figure** 1B, Figure S2D). Through unsupervised clustering, 17,393 cells were identified as MPCs and further divided into 25 subgroups (**Figure** 1B, **Table** S2). A heatmap shows the top five genes that are highly expressed in each MPC (**Figure** 1C). Based on the expression of representative genes such as Cd14, Ly6c, Ccr2, Csf1r, Adgre1, Cd209a, Itgax, MHCII, Lyve1, Ccl24, Timd4, CD45, Itgam, Ly6g, and MerTK, MPCs were categorized as heart resident macrophages (HRM), infiltrating monocyte-derived macrophages (IMs), monocytes, and DCs (**Figure** 1D, Figure S2E).

### S100a9^hi^ macrophages are acute inflammatory markers of MIR injury

**Figure 2:**
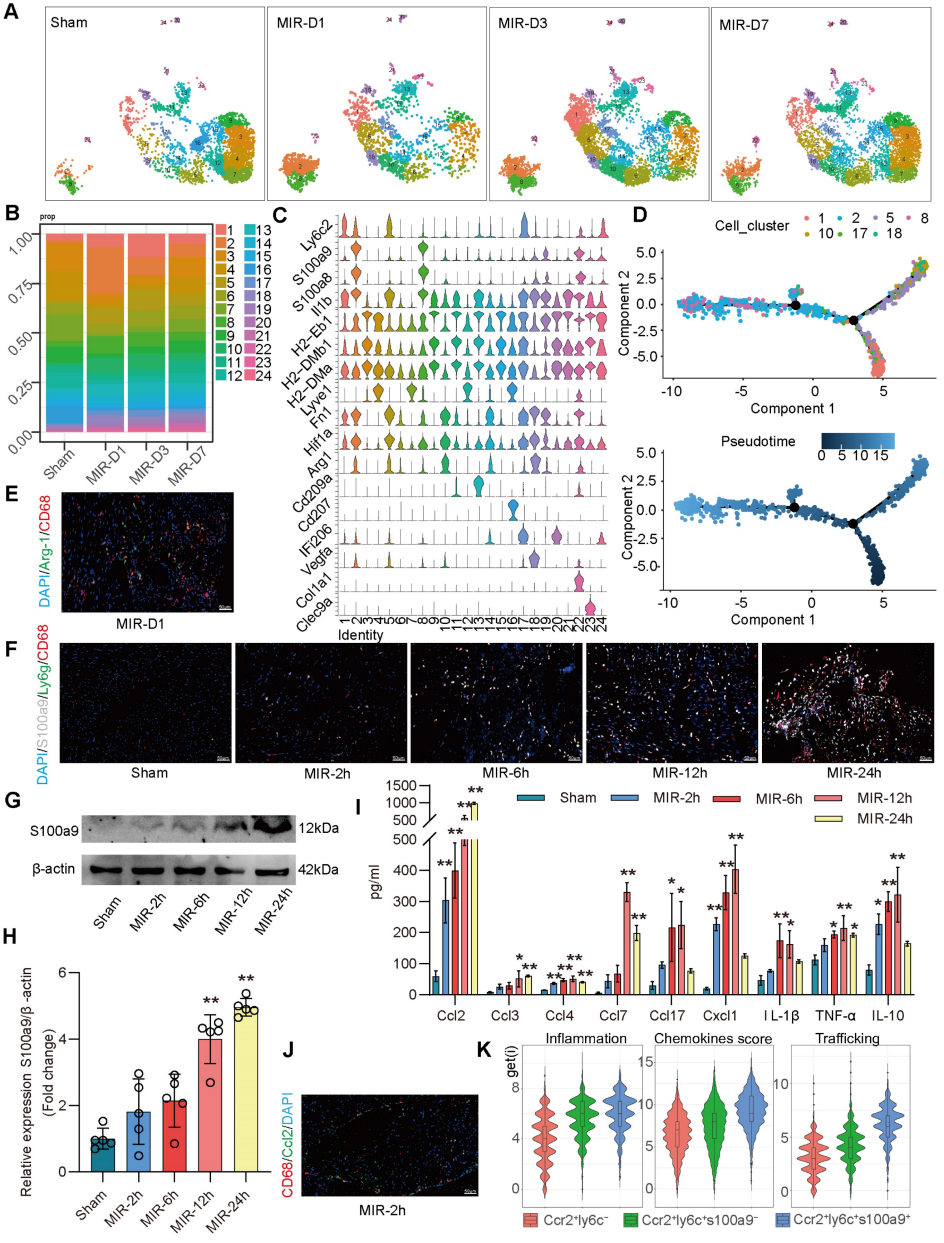
** S100a9^hi^ macrophages are the primary blood-derived inflammatory response cells after MIR.** (A) UMAP plots demonstrating the MPC cluster distribution at each time point in heart. (B) Fraction of each MPC population relative to all cardiac MPCs during the MIR progression. (C) Stacked violin plot of key genes in each MPC cluster. (D) Focused Monocle trajectory analysis including only the monocytes and monocyte-derived infiltrated macrophages in day 1 after MIR. (E) Representative immunofluorescence images of Arg-1 and CD68 co-staining of day 1 post injury heart tissues. n = 5. All scale bar, 50 μm. (F) Representative immunofluorescence images of CD68, S100a9, Ly6g co-staining of Sham or 2, 6, 12, 24 h post injury heart tissues. n = 3. All scale bar, 50 μm. (G-H) Western blots of S100a9 and the quantification of expression of Sham or 2, 6, 12, 24 h post injury heart tissues. n = 5. ns, no significance, *P < 0.05, **P < 0.01, compared to Sham, one-way ANOVA. (I) Quantification of cardiac chemokine concentrations at different time points after MIR. n = 3. ns, no significance, *P < 0.05, **P < 0.01, compared to Sham, one-way ANOVA. (J) Representative immunofluorescence images of Ccl2 and CD68 co-staining of 2 h post-MIR in heart tissues. All scale bar, 50 μm. n = 3. (K) Biological functional scores (Inflammation (left), Chemokines (middle), Trafficking (right)) of monocytes and monocyte-derived infiltrated macrophages.

Based on the proportions of MPCs in mouse hearts at different time points and the tSNE map, on day 1 after MIR, Clusters 2, 8 (S100a9^hi^ macrophages), 10, and 18 (Arg-1^hi^ macrophages) showed significant increases in the infiltration of monocyte-derived macrophages (**Figure** 2A-C). Clusters 1, 5, and 17 showed marked increases in monocytes. Pseudotime analysis revealed a transition from monocytes to Arg-1^hi^ macrophages, which subsequently transformed into S100a9^hi^ macrophages on day 1 post-MIR (**Figure** 2D). Immunofluorescence analysis confirmed the presence of Arg-1^hi^ and S100a9^hi^ macrophages in cardiac tissues on day 1 after MIR (**Figure** 2E-F, **Figure** S3A-B). H&E staining indicated that inflammatory cells began infiltrating cardiac tissue 2 h after MIR, peaking at 24 h (**Figure** S3C). Based on the reperfusion time points, the infiltration levels of S100a9^hi^ macrophages were assessed. The Sham group showed a negligible presence of S100a9^hi^ macrophages, but 2 h after MIR, S100a9^hi^ macrophages began infiltrating damaged cardiac tissue, peaking at 24 h post-reperfusion (**Figure** 2F, **Figure** S3B). Given that S100a9 is expressed in not only macrophages but also granulocytes, we examined the levels of S100a9^hi^ neutrophils at different times after MIR. 6 h after MIR, S100a9^hi^ granulocytes were observed, indicating that S100a9^hi^ macrophages infiltrated cardiac tissue before S100a9^hi^ neutrophils, serving as the primary blood-derived inflammatory responders (**Figure** 2F, **Figure** S3B). Protein analysis of S100a9 further validated the change in S100a9 after MIR (**Figure** 2G-H).

To explore the changes in cytokines driving acute-phase inflammatory cell infiltration post-reperfusion, mouse chemokines were examined, and 2 h after MIR, Ccl2, Ccl4, and Cx3cl1 levels began to increase, with Ccl2 showing the highest increase (**Figure** 2I, **Figure** S3D). This suggests that Ccl2 is the primary chemokine driving S100a9^hi^ macrophage infiltration into damaged myocardial tissue. Immunofluorescence analysis confirmed that the presence of macrophages was accompanied by significant expression of Ccl2 on 2 h after MIR (**Figure** 2J, **Figure** S3E).

In subsequent analyses, macrophages expressing Ccr2 were categorized based on Ly6c and S100a9 expression into three subgroups. Clusters 10 and 18 represented Ccr2^+^Ly6c^-^ macrophages, Clusters 1, 5, and 17 were Ccr2^+^Ly6c^+^S100a9^low^ macrophages, and Clusters 2 and 8 were Ccr2^+^Ly6c^+^S100a9^hi^ macrophages. The inflammatory score of Ccr2^+^Ly6c^+^ macrophages was significantly higher than that of Ccr2^+^Ly6c^-^ macrophages. The migration (transport capacity) and chemokine production biological scores of Ccr2^+^Ly6c^+^S100a9^hi^ macrophages were significantly higher than those of Ccr2^+^Ly6c^+^S100a9^low^ macrophages (**Figure** 2K).

### S100a9^hi^ Macrophages Mediate Acute Inflammatory Injury After MIR through the Myd88/NFκB/NLRP3 Signaling Pathway

**Figure 3:**
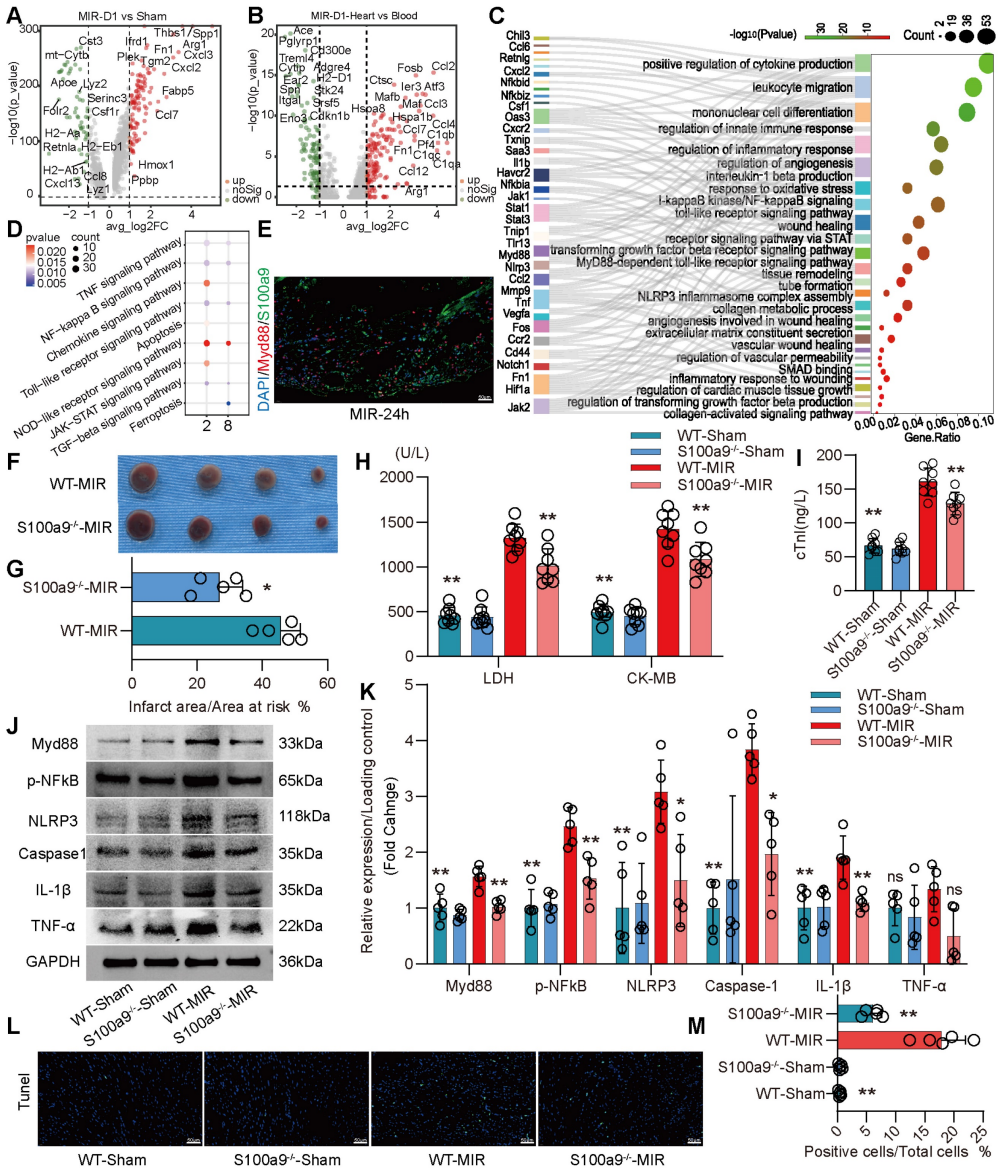
** S100a9^hi^ macrophages mediate and amplify the inflammatory response through the Myd88/NFκB/NLRP3 signaling pathway.** (A) Volcano plot of the fold-change in differential gene expression in day 1 post-MIR and Sham from all cells in the monocyte-derived infiltrated macrophages. (B) Volcano plot of the fold-change in differential gene expression in heart and peripheral blood from all monocytes of day 1 post-MIR.P-values were calculated by Wilcoxon rank sum test and P adjusted values were corrected by Bonferroni analysis. (C) Pathway Enrichment analysis of Cluster 2 based on the GO database. (D) KEGG pathway analysis of gene in the S100a9^hi^ macrophages with MetaboAnalyst 5.0. (E) Representative immunofluorescence images of Myd88 and S100a9 co-staining of 24 h post-MIR heart tissues. n = 3. All scale bar, 50 μm. (F-G) co-staining of Evans blue and TTC to detect infarct area and the quantification of infarct area. n = 5. ns, no significance, *P < 0.05, **P < 0.01, compared to WT-MIR, Student's t test. All scale bar, 50 μm. (H-I) The serum LDH, CK-MB, and cTnI level in Sham or MIR mouse model. n = 8 in each group. ns, no significance, * P < 0.05, ** P < 0.01 compared to WT-MIR group, one-way ANOVA. (J-K) Western blots of heart Myd88, phospho-NFκB, NLRP3, Caspase-1, IL-1β, and TNF-α and the quantification of expression on day 1 after MIR and Sham group. n = 5 in each group. ns, no significance, * P < 0.05, ** P < 0.01 compared to WT-MIR group, one-way ANOVA. (L-M) Representative images of Tunel assay of heart sections one day after treatment and quantitative analysis in each group. n = 5. ns, no significance, * P < 0.05, ** P < 0.01 compared to WT-MIR, one-way ANOVA. All scale bar, 50 μm.

We compared the differences in gene expression in monocyte-derived macrophages in heart tissues on the day 1 post MIR and in the Sham group. On the day 1 post MIR, monocyte-derived macrophages highly expressed inflammation-related genes, such as Spp1 and Hif1α; fibroskeletal repair genes, such as Fn1, Thbs1, and Plek; chemokines, such as Cxcl2 and Cxcl3; and complement genes, such as C1qa (**Figure** 3A). Compared to monocytes in the blood of mice on the day 1 post-reperfusion, monocytes in the heart highly expressed the chemokines Ccl2, Ccl3, Ccl4, and Ccl7, the hypoxia-ischemia stress-related genes Atf3, Hspa1a, and Hspa1b, and the complement genes C1qa, C1qb, and C1qc (**Figure** 3B). These changes facilitate chemotaxis and inflammation after monocyte infiltration into the heart after reperfusion.

On day 1 after MIR, S100a9^hi^ macrophages were highly enriched in genes associated with chemotaxis, leukocyte migration, monocyte differentiation, and the Myd88/NFκB/NLRP3 signaling pathway (**Figure** 3C-D, **Figure** S4A). We detected the colocalization of S100a9 and Myd88, verifying that S100a9 activated downstream Myd88 signaling (**Figure** 3E, **Figure** S4B). Subsequently, we constructed S100a9^-/-^ mice and established an MIR model. Compared to that in WT mice subjected to MIR, the infarct area in S100a9^-/-^ mice was significantly reduced (**Figure** 3F-G). Moreover, knockout of S100a9-gene effectively reduced the level of cardiac injury, decreasing the expression levels of CKMB, cTnI, and LDH (**Figure** 3H-I). We then measured the changes in protein levels in the Sham group and MIR mice. After MIR, the protein expression levels of p-NFκB, Myd88, and NLRP3 and the inflammatory mediators TNFα and IL-1β in WT mice were significantly higher than those in the Sham group (**Figure** 3J-K). Compared to WT mice, S100a9^-/-^ mice expressed lower levels of p-NFκB, Myd88, NLRP3, and inflammatory factors after MIR. The increased level of cell death in myocardial tissues post-reperfusion was alleviated in S100a9^-/-^ mice (**Figure** 3L-M, **Figure** S4C).

### S100a9^hi^ Macrophages Promote Fibrotic Remodeling through the Release of Tgf-β

**Figure 4:**
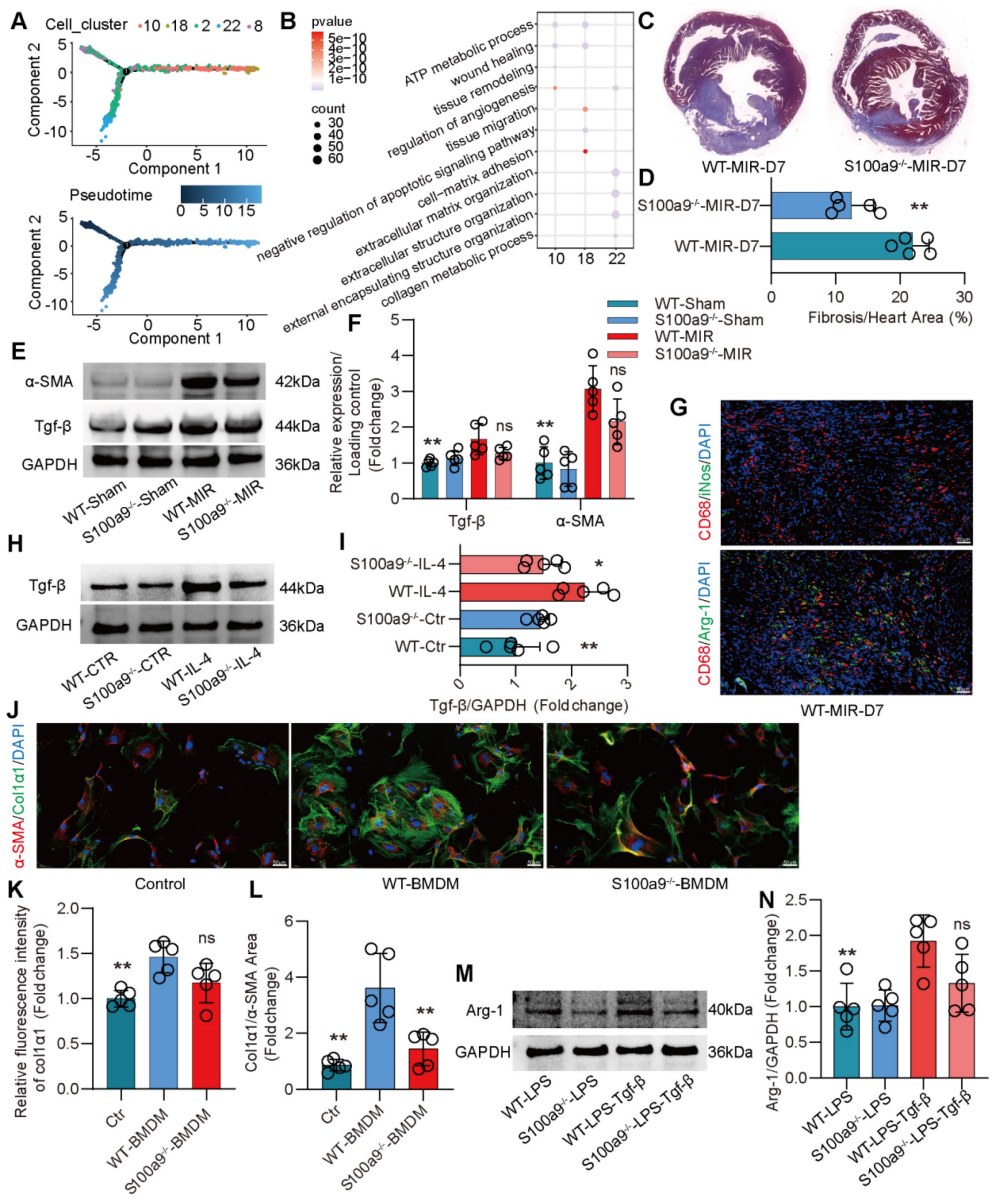
** S100a9^hi^ macrophages release Tgf-β to promote fibroblast activation into myofibroblasts, facilitating fibrotic remodeling.** (A) Focused Monocle trajectory analysis including monocyte-derived infiltrated macrophages in day 7 after MIR. (B) GO richment pathway analysis of gene in the Arg-1^hi^ and Col1α1^hi^ macrophages with MetaboAnalyst 5.0. (C-D) Representative images of Masson staining of heart sections on day 7 after MIR and quantitative analysis in each group. n = 5. * P < 0.05, ** P < 0.01 compared to WT-MIR, Student's t test. (E-F) Western blots of heart α-SMA, Tgf-β and the quantification of expression on day 7 after MIR and Sham group. n = 5 in each group. ns, no significance, * P < 0.05, ** P < 0.01 compared to WT-MIR group, one-way ANOVA. (G) Representative immunofluorescence images of iNos and CD68, as well as Arg-1 and CD68 co-staining of day 7 post-MIR in heart tissues. n = 5. All scale bar, 50 μm. (H-I) Western blots of Tgf-β and the quantification of expression of macrophage treated with IL-4 or not. n = 5 in each group. ns, no significance, * P < 0.05, ** P < 0.01 compared to WT-MIR, one-way ANOVA. (J-L) Representative immunofluorescence images of Col1α1 and α-SMA co-staining and quantitative analysis of releasing Col1α1 level in each group. n = 5. ns, no significance, * P < 0.05, ** P < 0.01 compared to WT-BMDMs, one-way ANOVA. All scale bar, 50 μm. (M-N) Western blots of Arg-1 and the quantification of expression in each group. n = 5 in each group. ns, no significance, * P < 0.05, ** P < 0.01 compared to WT-MIR, one-way ANOVA.

Compared to that on day 1 after MIR, the expression of macrophage chemotaxis and inflammatory genes in the heart gradually decreased on day 3 and 7 after MIR (**Figure** S5A-B). Based on the changes in macrophage populations at different time points, we observed that from day 3 to 7, the level of S100a9^hi^ macrophages gradually decreased, while Arg-1^hi^ and Col1α1^hi^ macrophages significantly increased (**Figure** 2A-B). Pseudotime analysis showed a transition from S100a9^hi^ macrophages to Arg-1^hi^ and Col1α1^hi^ macrophages (**Figure** 4A). GO enrichment analysis indicated that these three clusters of cells were mainly enriched in genes associated with tissue repair, injury repair, angiogenesis, and extracellular matrix generation (**Figure** 4B). Arg-1^hi^ macrophages have always been regarded as reparative M2 macrophages, promoting the activation of fibroblasts through the release of a large amount of Tgf-β. On day 7, significant fibrosis was observed in the heart, and the fibrotic area in S100a9^-/-^ mice was significantly reduced (**Figure** 4C-D). The protein level of α-SMA, a marker of myofibroblasts, verified that knockout of S100a9 could effectively reduce the activation of myofibroblasts, and the change in Tgf-β, an activator of myofibroblasts, was consistent with that of α-SMA (**Figure** 4E-F).

On day 7 after MIR, immunofluorescence analysis confirmed that macrophages mainly expressed Arg-1, representing the reparative M2 phenotype (**Figure** 4G, **Figure** S5C). Therefore, we extracted BMDMs from WT and S100a9^-/-^ mice, induced them to transform into the M2 phenotype, and explored the ability of these macrophages to release Tgf-β. We found that the level of Tgf-β released by BMDMs from S100a9^-/-^ mice was significantly lower than that released by BMDMs from WT mice (**Figure** 4H-I). BMDMs were induced to the M2 phenotype and cocultured with mouse PCFs. BMDMs from WT mice effectively promoted the transformation of fibroblasts into myofibroblasts, while the level of fibroblasts that transformed into myofibroblasts induced by macrophages from S100a9^-/-^ mice stimulated by IL-4 was reduced (**Figure** 4J-L, **Figure** S5D). To further explore the mechanism by which S100a9^hi^ macrophages transition from a highly inflammatory phenotype to the Arg-1^hi^ M2 phenotype after MIR, we stimulated the macrophages with LPS for 24 h and then stimulated them with Tgf-β for another 24 h. We found that macrophages from WT mice highly expressed Arg-1, while the level of Arg-1 expressed by S100a9^-/-^ macrophages was significantly decreased (**Figure** 4M-N).

### S100a9^hi^ Macrophages Promote Fibrosis through MMT

**Figure 5:**
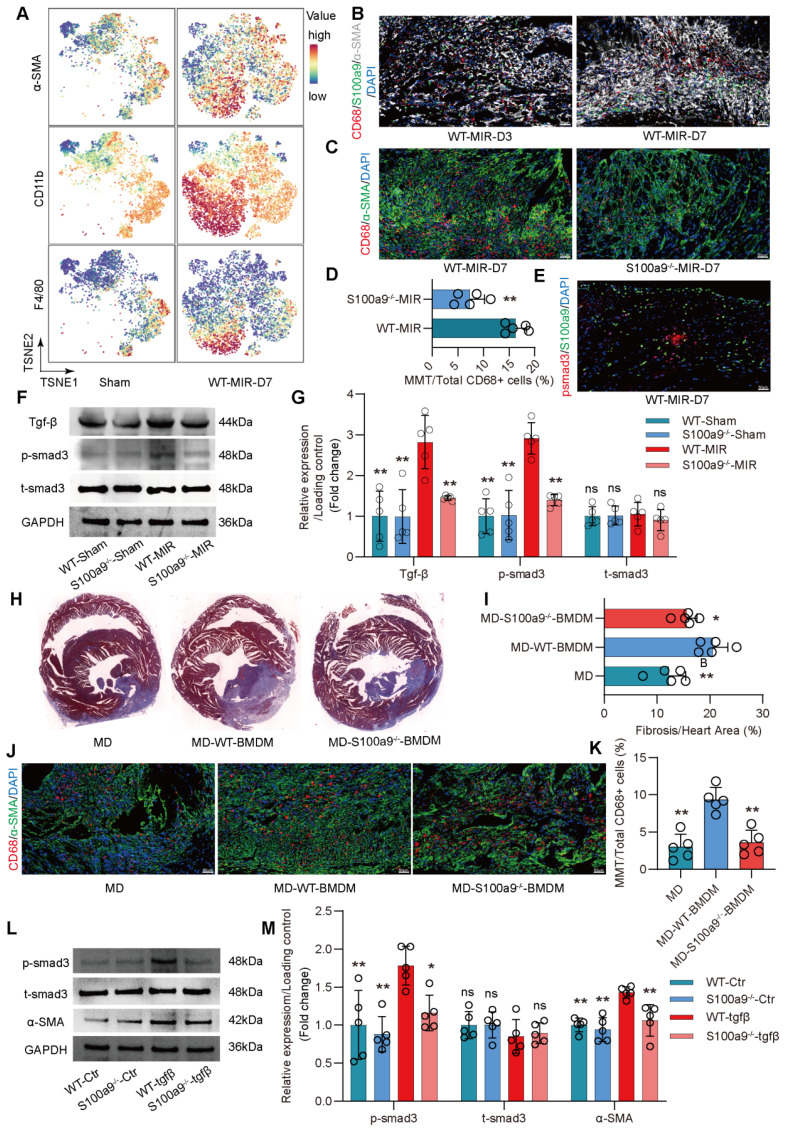
** S100a9^hi^ macrophages transition to myofibroblasts through the activation of the Tgf-β/smad3 signaling pathway.** (A) Representative TSNE plots from CyTOF data showing colored expression in arbitrary units (AU) of CD11b, F4/80, and α-SMA in cardiac macrophage in Sham and MIR group (n = 9). (B) Representative immunofluorescence images of S100a9, CD68, and α-SMA co-staining of day 3 and day 7 post injury heart tissues. n = 5 in each group. All scale bar, 50 μm. (C-D) Representative immunofluorescence images of CD68 and α-SMA co-staining and quantitative analysis in each group. n = 5. ns, no significance, * P < 0.05, ** P < 0.01 compared to WT-MIR, Student's t test. All scale bar, 50 μm. (E) Representative immunofluorescence images of p-smad3 and S100a9 co-staining of day 7 post injury heart tissues. n = 5 in each group. All scale bar, 50 μm. (F-G) Western blots of heart Tgf-β, p-smad3, t-smad3, and the quantification of expression on day 7 after MIR and Sham group. n = 5 in each group. ns, no significance, * P < 0.05, ** P < 0.01 compared to WT-MIR group, one-way ANOVA. (H-I) Representative images of Masson staining of heart sections and quantitative analysis in each group. n = 5. ns, no significance, * P < 0.05, ** P < 0.01 compared to MD group, one-way ANOVA. (J-K) Representative immunofluorescence images of CD68 and α-SMA co-staining and quantitative analysis in each group. n = 5. ns, no significance, * P < 0.05, ** P < 0.01 compared to MD, one-way ANOVA. All scale bar, 50 μm. (L-M) Western blots of α-SMA, p-smad3, t-smad3, and the quantification of expression in each group. n = 5 in each group. ns, no significance, * P < 0.05, ** P < 0.01 compared to WT-Tgfβ group, one-way ANOVA.

Cd45^+^Cd11b^+^ cells were isolated from hearts in the Sham group and MIR group on day 7 and subjected to mass cytometry. Initial clustering revealed a cellular profile composed of fibroblasts and a significant number of immune cells (**Figure** S6A). Macrophages were further clustered into 12 subsets. Compared to that in the Sham group, on day 7 after MIR, there was an increase in the presence of macrophages coexpressing high levels of macrophage and myofibroblast markers (**Figure** 5A). This result confirmed that MMT occurred in damaged heart tissue after MIR. Immunofluorescence staining indicated significant macrophage infiltration in fibrotic tissues after MIR, and S100a9^hi^ macrophages expressed the myofibroblast marker α-SMA (**Figure** 5B, **Figure** S6B). These S100a9^+^CD68^+^α-SMA^+^ cells appeared as early as day 3 post-reperfusion, suggesting concurrent inflammation and repair processes. On day 7 post-reperfusion, fibrosis and macrophage levels in cardiac tissues were assessed. S100a9^-/-^ mice showed reduced macrophage numbers and fibrosis levels compared to WT mice. MMT levels in S100a9^-/-^ mice were also lower than those in WT mice (**Figure** 5C-D, **Figure** S7A). Tgf-β/p-smad3 was identified as a key mechanism that affected MMT, and violin plots confirmed higher expression levels of smad3 and α-SMA in S100a9^hi^ cells than in other macrophage subsets (**Figure** S7B). Immunofluorescence analysis confirmed the colocalization of S100a9 and p-smad3 (**Figure** 5E, **Figure** S7C). On day 1 after MIR, p-smad3 levels in WT and S100a9^-/-^ mice were assessed, and knockout of S100a9 effectively reduced p-smad3 levels, which was consistent with the changes in Tgf-β levels (**Figure** 5F-G).

Subsequent experiments involved depleting macrophages in mice using liposomes 24 h before establishing the MIR model. Compared to WT mice, macrophage-depleted mice showed significant reductions in myocardial fibrosis after MIR (**Figure** 5H-I). The expression of α-SMA and CD68 was also significantly reduced. To explore whether S100a9^hi^ macrophages could promote fibrosis progression, Mice with MD were reinfused with BMDMs from WT and S100a9^-/-^ mice on day 4 after MIR.

Compared to regular mice with MD, mice with MD reinfused with WT mice-derived BMDMs showed significant exacerbation of myocardial fibrosis (**Figure** 5J-K, **Figure** S7D). However, the increase in fibrosis in mice with MD infused with S100a9^-/-^ BMDMs was slightly lower than that in mice infused with WT mice-derived BMDMs. Consistent with the changes in fibrosis, MMT levels were highest in mice infused with WT mice-derived BMDMs, while infusion with S100a9^-/-^ mice-derived BMDMs did not significantly affect the increase in MMT. After depleting macrophages, there was no significant difference in Ly6g^+^ neutrophils, regardless of whether exogenous BMDM was reinfused (**Figure** S7 E-F).

In vitro, BMDMs from WT mice were cultured, and after 5 days of Tgf-β stimulation, these cells showed high expression of α-SMA, confirming MMT in macrophages (**Figure** S8A-B). MMT was accompanied by a significant increase in p-smad3 levels. BMDMs from S100a9^-/-^ mice showed significantly reduced α-SMA expression compared to WT mice-derived BMDMs, which was accompanied by a reduction in p-smad3 levels (**Figure** 5L-M). At day 30 post-reperfusion, cardiac function in mice was assessed. S100a9-gene deficiency tend to reverse the decline in cardiac function caused by MIR and reduced the cardiac index (**Figure** S8C-E).

### S100a9 is an Effective Target for Alleviating Inflammatory Injury and Fibrotic Remodeling after MIR

**Figure 6:**
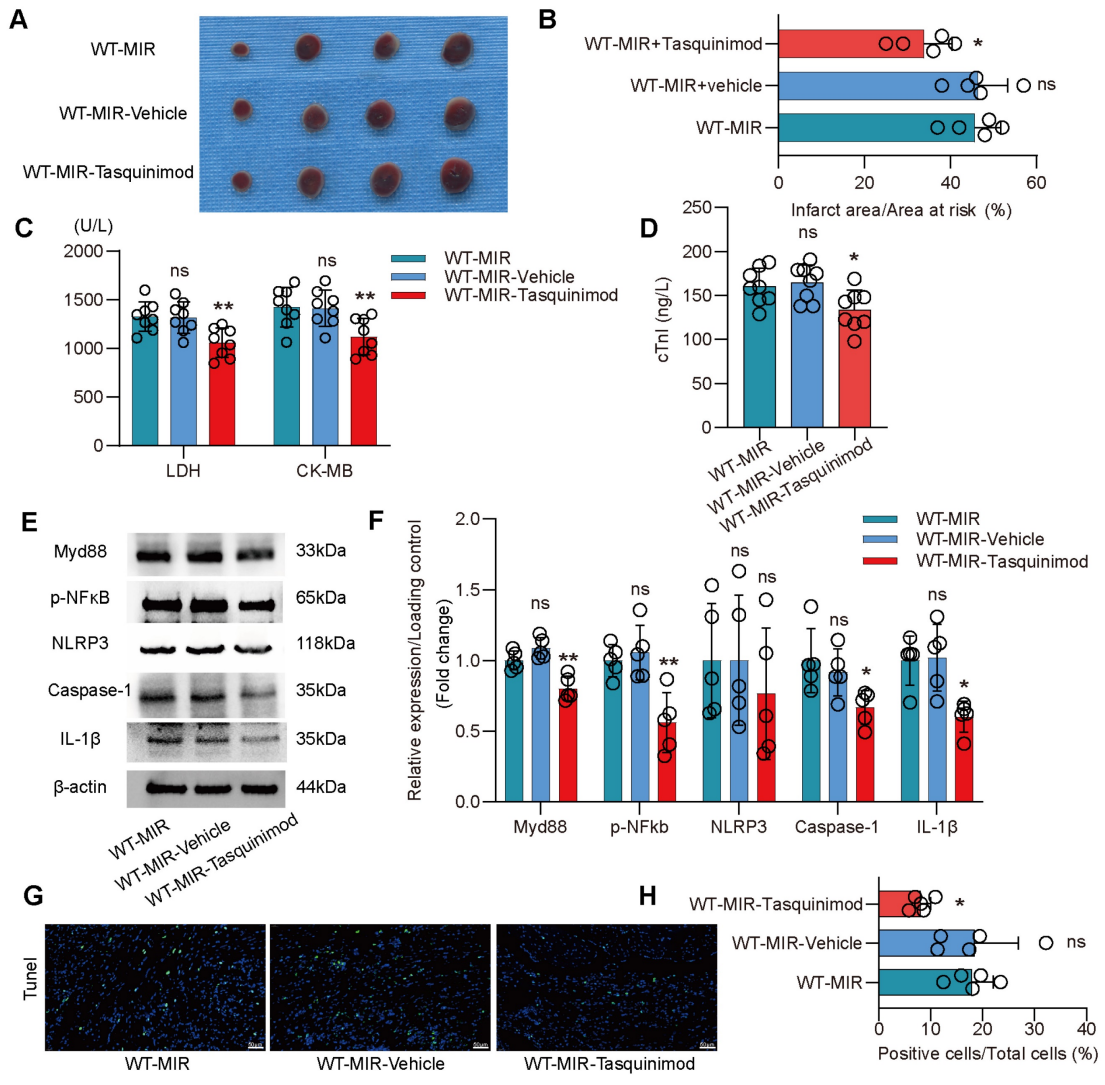
** Pharmacological inhibition of S100a9 alleviates acute inflammatory damage after MIR.** (A-B) co-staining of Evans blue and TTC to detect infarct area and the quantification of infarct area. n = 5. ns, no significance, *P < 0.05, **P < 0.01, compared to WT-MIR, one-way ANOVA. (C-D) The serum LDH, CK-MB, and cTnI level in MIR mouse model. n = 8 in each group. ns, no significance, * P < 0.05, ** P < 0.01 compared to WT-MIR group, one-way ANOVA. (E-F) Western blots of heart Myd88, phospho-NFκB, NLRP3, Caspase-1, IL-1β, and the quantification of expression on day 1 after MIR. n = 5 in each group. ns, no significance, * P < 0.05, ** P < 0.01 compared to WT-MIR, one-way ANOVA. (G-H) Representative images of Tunel assay of heart sections at day 1 after treatment and quantitative analysis in each group. n = 5. ns, no significance, * P < 0.05, ** P < 0.01 compared to WT-MIR, one-way ANOVA. All scale bar, 50 μm.

To validate S100a9 as a therapeutic target for inflammatory injury and fibrotic remodeling after MIR, we used tasquinimod, a selective S100a9 inhibitor. Compared to the MIR group, the solvent control group showed almost no significant changes. However, the S100a9 inhibitor significantly reduced the myocardial infarction area after MIR and reduced the levels of myocardial injury markers (**Figure** 6A-D). Analysis of the Myd88/NFκB/NLRP3 signaling pathway revealed that the S100a9 inhibitor significantly reduced the inflammatory response mediated by Myd88/NFκB/NLRP3 (**Figure** 6E-F). Tunel fluorescence analysis confirmed that inhibiting S100a9 reduced cell death after MIR (**Figure** 6G-H, **Figure** S9A).

**Figure 7:**
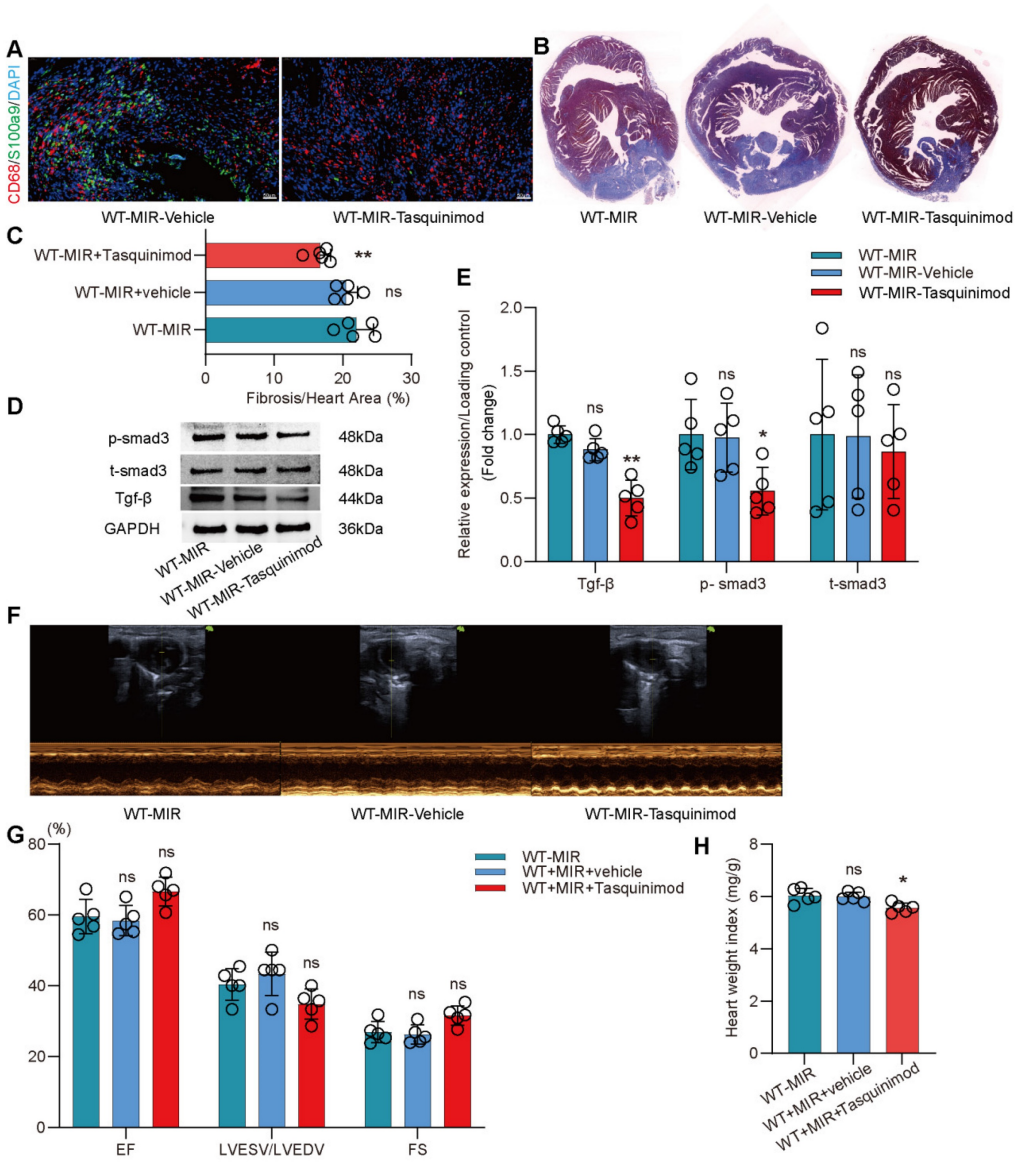
** Pharmacological inhibition of s100a9 effectively improves myocardial fibrotic remodeling and cardiac function after MIR.** (A) Representative immunofluorescence images of CD68 and S100a9 co-staining and quantitative analysis in each group. n = 5. ns, no significance, * P < 0.05, ** P < 0.01 compared to WT-MIR, one-way ANOVA. All scale bar, 50 μm. (B-C) Representative images of Masson staining of heart sections and quantitative analysis in each group. n = 5. ns, no significance, * P < 0.05, ** P < 0.01 compared to WT-MIR group, one-way ANOVA. (D-E) Western blots of heart Tgf-β, p-smad3, t-smad3, and the quantification of expression on day 7 after MIR. n = 5 in each group. ns, no significance, * P < 0.05, ** P < 0.01 compared to WT-MIR group, one-way ANOVA. (F-G) Echocardiographic analysis of LV end-diastolic volume (LVEDV)/LV end-systolic volume (LVESV), and LV ejection fraction (EF) at day 30 after MIR or Sham in WT and S100a9^-/-^ mice. n = 5 in each group. ns, no significance, * P < 0.05, ** P < 0.01 compared to WT-MIR group, one-way ANOVA. (H) Heart weight index at day 30 after MIR. n = 5 in each group. ns, no significance, * P < 0.05, ** P < 0.01 compared to WT-MIR group, one-way ANOVA.

The inhibitor significantly reduced the number of S100a9^hi^ macrophages in injured myocardial tissue (**Figure** 7A, **Figure** S9B). To explore the effect of inhibiting S100a9 on myocardial fibrotic remodeling, we continuously administered intraperitoneal injections of the inhibitor for one week and assessed myocardial fibrosis levels in the mice. Masson staining suggested that the S100a9 inhibitor effectively alleviated myocardial fibrosis (**Figure** 7B-C). We assessed Tgf-β/p-smad3 signaling and found that inhibiting S100a9 effectively suppressed the protein expression of Tgf-β/p-smad3, thereby reducing the activation of myofibroblasts and the release of collagen fibers (**Figure** 7D-E). At day 30 post-reperfusion, we assessed cardiac function in mice and found that the S100a9 inhibitor effectively improved the decline in cardiac function caused by MIR and increased the cardiac index (**Figure** 7F-H).

## Discussion

ScRNA-seq can effectively distinguish the heterogeneity of immune cells, elucidating the functional characteristics of different cell subtypes. The immune microenvironment in MI is exceedingly complex. Previous studies have shown the dynamic changes in immune cells after MI, highlighting the abundance and functional variations in immune cells 22-24. Monocyte-derived macrophages have been proven to play a significant role in damage and repair processes following MI. However, due to the focus on sequencing analysis of CD45^+^ immune cells, there has been reduced capture of macrophage abundance in the cell population, potentially overlooking the comprehensiveness of macrophage subgroup clustering. If the number of cells captured by scRNA-seq is too low, the subdivision of subgroups lacks biological significance. Based on their origin in the heart, macrophages are classified as HRM and infiltrative macrophages. A study by Dick et al. combined monocyte-macrophage sorting with scRNA-seq and confirmed that HRMs effectively protected cardiac function post MI and limited ventricular remodeling 25. However, for IMs, there has been a lack of comprehensive and systematic research exploring their dynamic functions and subgroup development post-MI. This study used markers such as CD45, CD11b, and Ly6c and sorted single nucleated cells to achieve maximum abundance. Building on these studies, we further analyzed the dynamic changes and functional mechanisms of IMs in myocardial injury and fibrosis.

In this study, we isolated monocytes based on the characteristic markers of traditional mononuclear macrophages, such as CD45, CD11b, and Ly6c. By combining mass cytometry and scRNA-seq, we showed the significant role of infiltrating monocyte-derived macrophages in the transition from the acute inflammatory phase to fibrotic repair after MIR. Our novel findings include the following: (1) S100a9^hi^ macrophages are the primary blood-derived source of inflammatory response cells with key functions in initiating MIR injury; (2) S100a9 activates sterile inflammatory responses through the Myd88/NFκB/NLRP3 signaling pathway; and (3) S100a9^hi^ macrophages can transform into the reparative M2 phenotype to promote fibroblast activation and activate Tgf-β/p-smad3-mediated MMT to exacerbate fibrotic progression.

Macrophages, which are crucial factors in innate immunity, play an irreplaceable role in MIR injury 26. scRNA-seq revealed a substantial increase in macrophage abundance 24 h post-reperfusion. Based on the marker genes of HRMs (including Timd4, Lyve1, Ccl24, Cd163, and MHC-II) and Ly6c and Ccr2, macrophages were classified as HRMs and IMs. On the day 1 post-reperfusion, the majority of enriched macrophages in the heart originated from bone marrow-derived monocytes, while the level of HRMs significantly decreased, indicating the important role of IMs in MIR. Based on pseudotime analysis, we found that infiltrating monocytes primarily differentiated into S100a9^hi^ macrophages 24 h post-reperfusion. These macrophages highly express inflammatory genes, chemokines, and inflammatory mediators and are enriched in the Myd88/NFκB/NLRP3 signaling cascade to amplify inflammatory responses. As early as 2 h after MIR, damaged cardiac tissues released large amounts of chemokines, such as Ccl2, Ccl4, and Cxcl1, coinciding with the infiltration of S100a9^hi^ macrophages. Inflammatory mediators such as IL-1β and TNF-α peaked at 12 h. S100a9 has been regarded as a reliable biomarker of inflammation. In the acute phase of MI, S100a9 is rapidly released at the site of ischemic injury and is increased in the coronary artery and systemic circulation before myoglobin and troponin 27. Elevated levels of S100a9 after percutaneous coronary intervention (PCI) can predict the incidence of major adverse cardiovascular events (MACEs) in AMI patients 28. Since S100a9 is highly expressed in not only macrophages but also neutrophils, we examined the changes in levels of neutrophils after MIR and found that neutrophils began to infiltrate damaged cardiac tissue at 6 h. This further confirmed that S100a9^hi^ macrophages are the primary inflammatory cells in the acute phase of MIR. Functionally, in the acute phase, S100a9^hi^ macrophages mainly form inflammasomes via the Myd88/NFκB/NLRP3 signaling cascade, further releasing inflammatory mediators such as IL-1β to promote acute sterile inflammatory responses. S100a9-gene deficiency significantly inhibited the activation of Myd88/NFκB/NLRP3 signaling and alleviated acute MIR injury.

After acute MIR injury, due to severe ischemia and hypoxia, the environment characterized by oxidative stress and inflammatory injury gradually changes to a reparative microenvironment. Cells in the tissue release large amounts of functional cytokines to promote angiogenesis and tissue remodeling. Arg-1^hi^ macrophages infiltrate the heart on the day 1 post MIR, further indicating that repair and inflammatory injury are simultaneous biological processes, and acute inflammation is predominant on the day 1 29. From the day 3 to the day 7 after MIR, macrophages mainly shift to the reparative M2 phenotype, and the abundance of Arg-1^hi^ macrophages and Col1α1^hi^ macrophages gradually increases. The transformation of S100a9^hi^ macrophages to M2 macrophages in the later stage shifts their proinflammatory functions to repair damaged tissues and increase angiogenesis.

Previous study has confirmed that short-term administration of S100a9 inhibitors can effectively reduce myocardial injury and protect heart function, but long-term administration of S100a9 inhibitors is harmful to cardiac contraction function 30. However, another study constructed S100a9^-/-^ mice and S100a9^Tg^ mice, and confirmed that compared to WT mice, the myocardial fibrosis area and myocardial contraction dysfunction of S100a9^-/-^ mice after MIR were significantly reduced, and the results observed in S100a9^Tg^ mice were completely opposite 28. This is consistent with the results we observed in our research. Interestingly, another study used rS100a8/a9 to treat MIR mice, which further promoted adverse myocardial remodeling, leading to extensive replacement fibrosis, and enhanced the myocardial expression of the important medium Tgf-β in remodeling 31. However, the specific mechanism of these results needs further exploration.

Previous research has shown that S100a9 can activate nur77, which in turn promotes the transformation of inflammatory macrophages into reparative macrophages 32. Interestingly, the activation and expression of Nur77 are mediated by Tgf-β-induced smad3 signaling and the transcription factor SP1 33. Temporarily upregulated Tgf-β can induce NR4A1 expression, thereby producing a negative feedback loop, but the persistent activation of Tgf-β signaling in fibrotic diseases will suppress the expression and activation of NR4A1 through AKT and HDAC dependent mechanisms 30. In this study, we found that Tgf-β increased during acute inflammation of reperfusion, and reached a peak on the day 7 post-MIR.

Tgf-β has always been regarded as the main cytokine that promotes the transformation of fibroblasts into myofibroblasts, and this factor mainly originates from macrophages. Previous studies have shown that the release of Tgf-β by macrophages depends on S100a9 34. Our research further confirmed these conclusions. On the day 1 and 7 post-reperfusion, the high expression of Tgf-β in the hearts of WT mice could be effectively alleviated in S100a9^-/-^ mice, which was accompanied by a reduction in the area of myocardial fibrosis. In vitro experiments also confirmed that S100a9-definciency macrophages had a weaker ability to promote the transformation of fibroblasts into myofibroblasts after IL-4 induction than WT mice-derived macrophages.

Beyond the activation of fibroblasts by Tgf-β to form myofibroblasts, macrophages derived from monocytes exhibit a high degree of bone marrow lineage plasticity and are capable of transitioning into myofibroblasts 35. Previous studies have identified populations of macrophages resembling fibroblasts in the hearts of mice on the day 7 post-MI 36. Haider. et al. observed that macrophages expressing YFP acquired fibroblast-like phenotypes during MI healing 20. However, these studies only confirmed that the highly fibrotic microenvironment after MI induces monocyte-derived macrophages into fibroblast-like phenotypes but did not determine the inherent mechanisms of these phenotypic transformations.

In organs exhibiting similar phenotypic transformations, Tgf-β/p-smad3 has been established as a pivotal mechanism in MMT 19, 37. In this study, compared to other macrophage types, S100a9^hi^ macrophages exhibited elevated expression of smad3 and α-SMA, suggesting the potential for MMT. On the day 1 after MIR, a significant increase in Tgf-β/p-smad3 was observed, and by the day 3, Cd68^+^S100a9^+^α-SMA^+^ cells were detectable, further confirming the activation of MMT during acute inflammation. Our results confirmed that S100a9 can mediate and amplify inflammation through Myd88/NFκB/NLRP3 during the acute period, and as the tissue microenvironment changes during the reperfusion process, it gradually transitions to M2 reparative macrophages, promoting the progression of fibrosis. This may be the positive feedback of Tgf-β leading to S100a9^hi^ macrophage transition from pro-inflammatory to pro-reparative macrophages, and it also confirms that fibrosis and inflammation are simultaneous processes, which is in line with the research conclusions of predecessors 30. S100a9-gene deficiency effectively reduced fibrosis levels following MIR. The Tgf-β/p-smad3 signaling cascade was notably inhibited after S100a9-gene knockout, and there were marked decreases in macrophage levels and the number of Cd68^+^α-SMA^+^ cells within damaged myocardial tissue. However, excessive inhibition of macrophage infiltration is not always beneficial for myocardial tissue repair. Depletion of macrophages led to a significant reduction in Tgf-β, thereby improving myocardial fibrosis. However, ruptured myocardial fibers or dead myocardial cells are inadequately filled and connected by the extracellular matrix, which is detrimental to the restoration of normal cardiac structure and function. The infusion of exogenous macrophages markedly exacerbated fibrosis and cells undergoing MMT, while the infusion of S100a9^-/-^ macrophages had a weaker impact on fibrosis and changes in MMT. Evidently, MMT mediated by S100a9^hi^ macrophages effectively participates in fibrotic alterations after MIR.

Our research confirms the role of S100a9^hi^ macrophages in inflammatory damage and fibrosis following MIR. Gene knockout can effectively alleviate myocardial inflammatory damage and fibrosis. Further use of tasquinimod in this study effectively improved myocardial inflammatory damage and chronic fibrotic remodeling. Tasquinimod has been considered an orphan drug for treating multiple myeloma, and several ongoing phase III clinical trials are underway for prostate cancer 38. Our findings provide compelling evidence for the clinical research on the use of tasquinimod to treat MI, and further large-scale clinical trials for cardiovascular applications are needed.

## Conclusion

This research comprehensively presents the dynamic single-cell atlas of MPCs after MIR, elucidating the origin, functional plasticity, and mechanism of infiltrating monocyte-derived macrophages during acute inflammation after MIR. Our study identifies a unique Ccr2^+^Ly6c^+^S100a9^hi^ macrophage population that activates the Myd88/NFκB/NLRP3 inflammatory signaling cascade, thereby initiating acute sterile inflammation after MIR. Furthermore, through the Tgf-β/p-smad3 signaling cascade, these cells promote MMT and fibroblast activation, facilitating myocardial fibrotic remodeling.

## Supplementary Material

Supplementary figures and table legends.

Supplementary table 1.

Supplementary table 2.

## Abbreviations

MIR: myocardial ischemia-reperfusion
Tgf-β: transforming growth factor-β
MMT: macrophage-to-myofibroblast transition
MI: acute myocardial infarction
scRNA-seq: single-cell RNA-sequencing
PBMC: Peripheral blood mononuclear cell
TTC: 2,3,5-Triphenyltetrazolium chloride
LDH: Lactate Dehydrogenase
cTnI: cardiac Troponin I
CKMB: Creatine Kinase Myocardial Band
H&E: hematoxylin-eosin
BMDM: Bone marrow-derived macrophage
M-CSF: macrophage-stimulating factor
IL-4: Interleukin 4
PCF: Primary cardiac fibroblast
WT: wild-type
MD: macrophage depletion
LVEF: left ventricular ejection fraction
FS: fractional shortening
LVESV/LVEDV: left ventricular end-systolic volume/left ventricular end-diastolic volume
MPC: mononuclear phagocytic cells
HRM: heart resident macrophages
IMs: infiltrating monocyte-derived macrophages
PCI: percutaneous coronary intervention
MACE: major adverse cardiovascular events
