# Supplementary material for: Single-cell RNA sequencing reveals S100a9hi macrophages promote the transition from acute inflammation to fibrotic remodeling after myocardial ischemia‒reperfusion
Source: Theranostics. 2024 Jan 20;14(3):1241–59. doi: 10.7150/thno.91180 (PMC10845204; doi:10.7150/thno.91180)
Supplement: Supplementary file 1 — Supplementary figures and table legends. [file thnov14p1241s1.pdf]

Figure S1

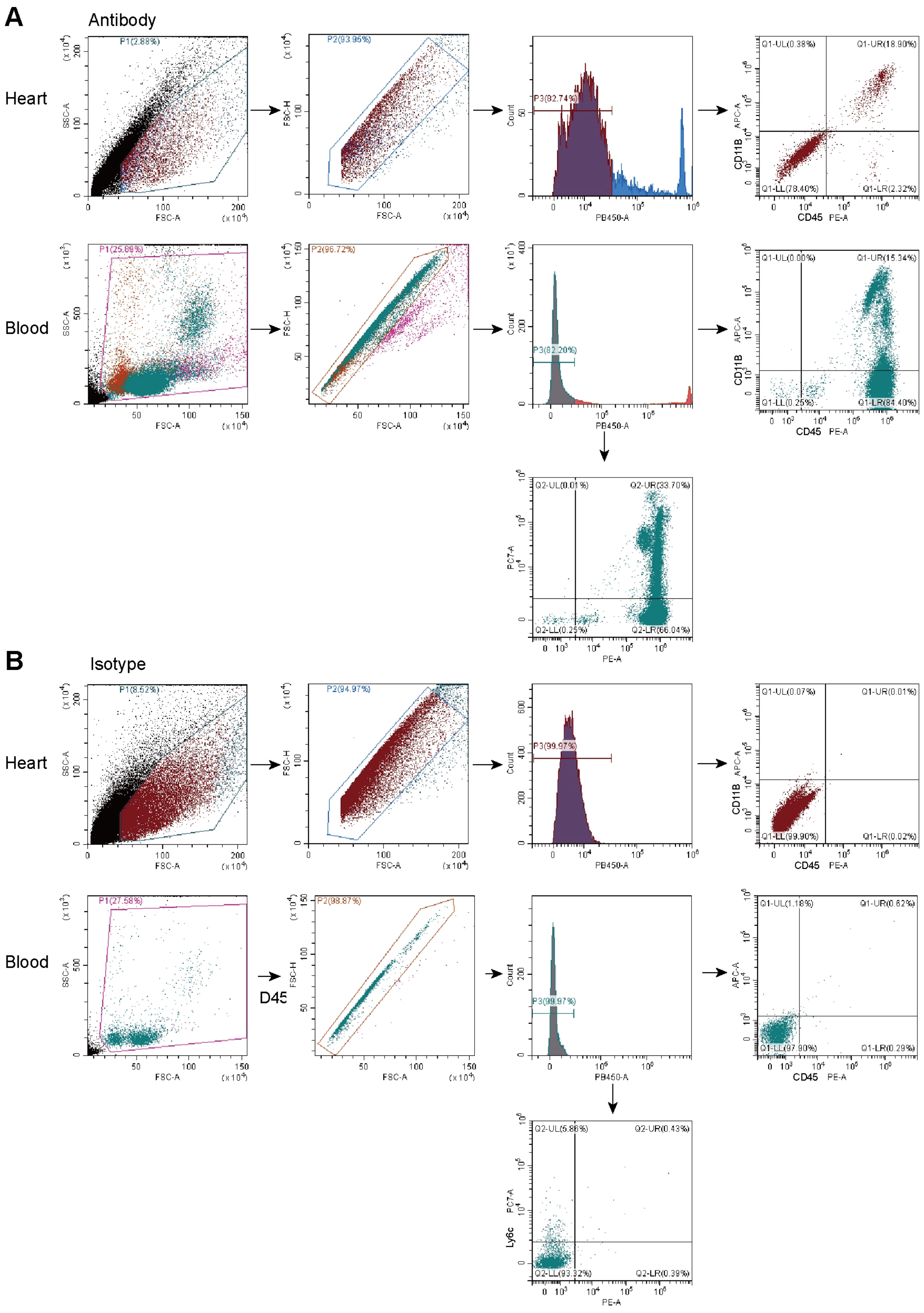
A
Antibody
Heart
Blood
B
Isotype
Heart
Blood
D45
P1(2.88%)
P2(93.95%)
P3(82.74%)
Q1-UL(0.38%)
Q1-UR(18.90%)
Q1-LL(78.40%)
Q1-LR(2.32%)
P1(25.89%)
P2(96.72%)
P3(82.20%)
Q1-UL(0.00%)
Q1-UR(15.34%)
Q1-LL(0.25%)
Q1-LR(84.40%)
Q2-UL(0.01%)
Q2-UR(33.70%)
Q2-LL(0.25%)
Q2-LR(66.04%)
P1(8.52%)
P2(94.97%)
P3(99.97%)
Q1-UL(0.07%)
Q1-UR(0.01%)
Q1-LL(99.90%)
Q1-LR(0.02%)
P1(27.58%)
P2(98.87%)
P3(99.97%)
Q1-UL(1.18%)
Q1-UR(0.62%)
Q1-LL(97.90%)
Q1-LR(0.29%)
Q2-UL(5.86%)
Q2-UR(0.43%)
Q2-LL(93.32%)
Q2-LR(0.39%)
SSC-A
FSC-A
FSC-H
Count
PB450-A
CD11B APC-A
CD45 PE-A
PC7-A
Ly6c PC7-A
PE-A

Figure S1. Sorting strategy of MPCs in MIR animals. (A) Representative flow cytometry gating strategies of MPCs collected from heart and blood at each time point. (B) Isotype control for flow cytometry experiment in A.

Figure S2

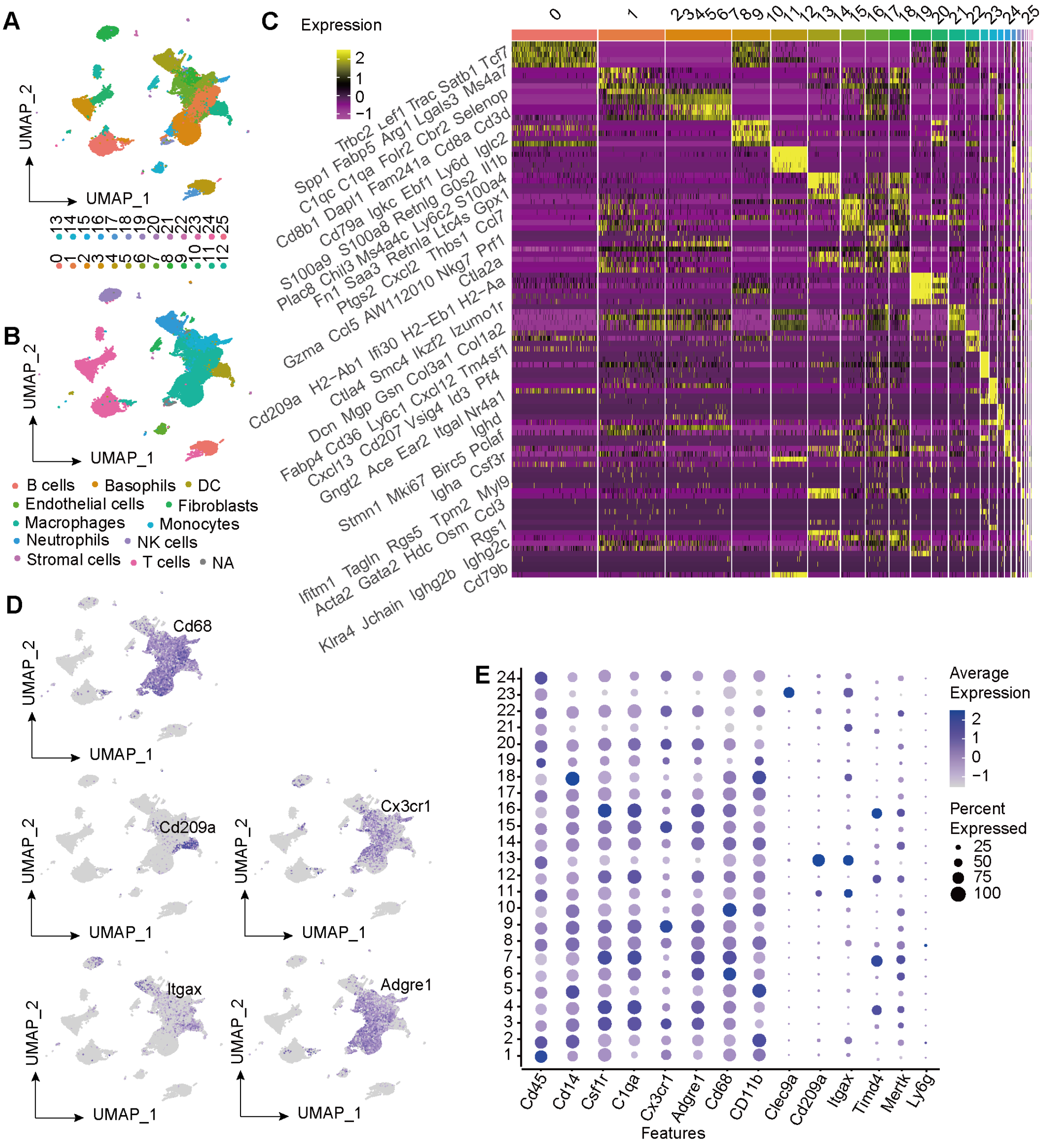

Figure S2. Identification of MPC populations in scRNA sequencing data of sorted cells from heart and blood. (A-B) UMAP plot of a total of 38497 cells after quality-control. (C) Heatmap showing the top 5 genes of each cluster. (D) Feature plots of MPC markers. (E) Dot plot of monocyte, macrophage and DCs markers in each MPC cluster.

Figure S3

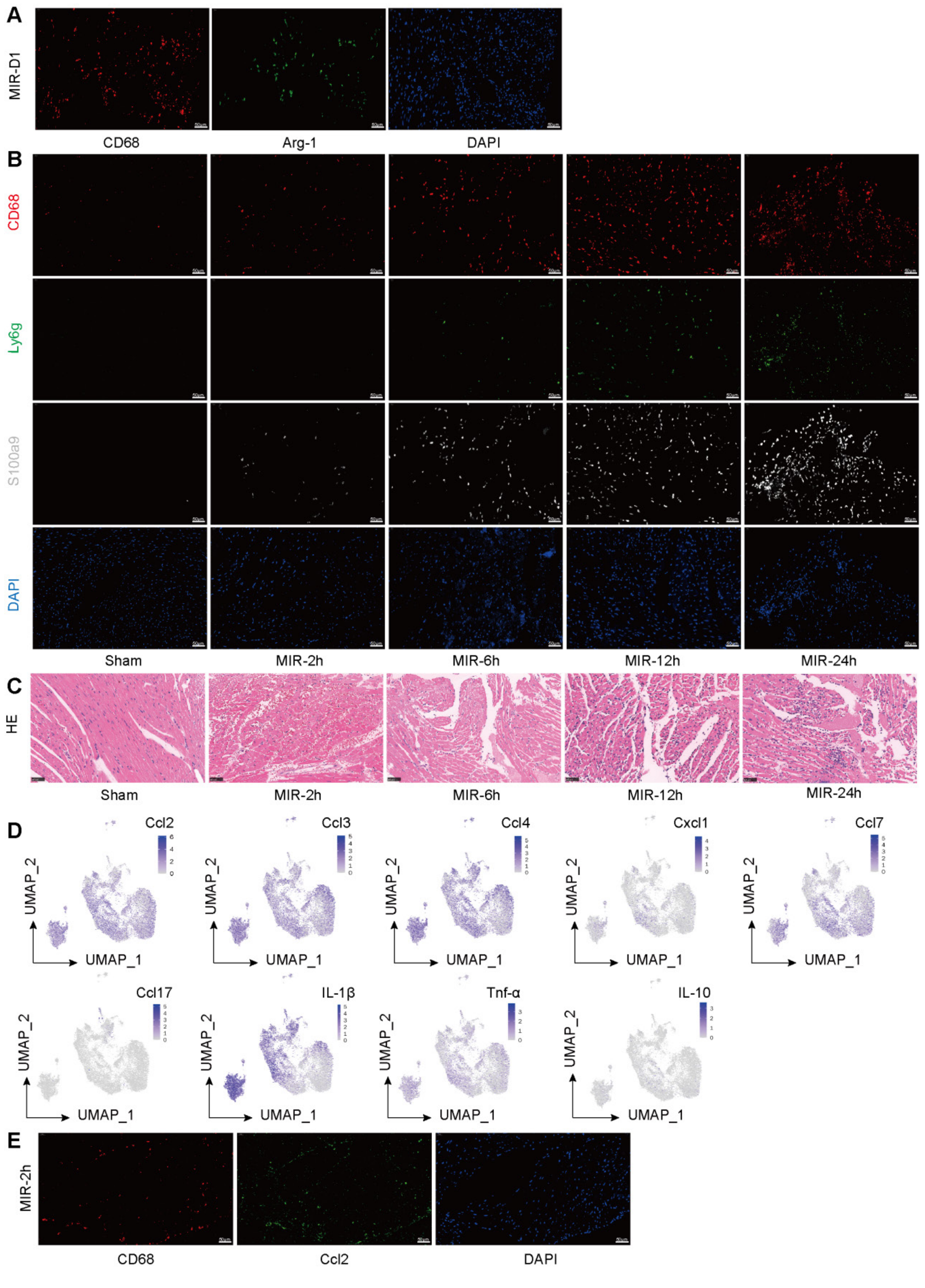

Figure S3: Trends in inflammatory macrophage infiltration at different time points within 24 h after MIR. (A) Images of the individual antibody staining for CD68 and Arg-1 in Figure 2E. n = 5. Scale bar, 50 μm. (B) Images of the individual antibody staining for CD68, S100a9 and Ly6g in Figure 2F. n = 3. Scale bar, 50 μm. (C) Representative images of heart H&E staining at each time point before and after MIR. n = 5. Scale bar, 50 μm. (D) UMAP plots demonstrating the key chemokine in

MPCs. (E) Images of the individual antibody staining for CD68 and CCL2 in Figure 2J. n = 3. Scale bar, 50 μm. MIR, myocardial ischemia reperfusion.

Figure S4

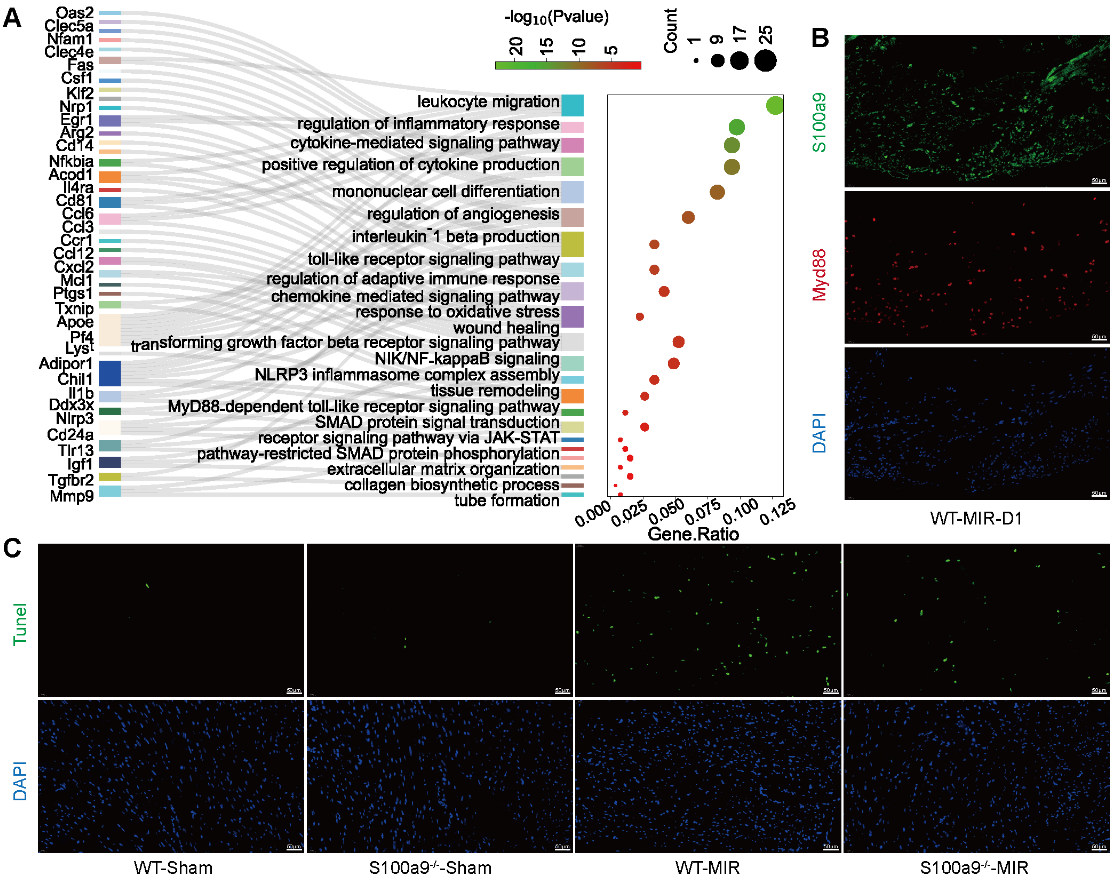

Figure S4: S100a9^{hi} macrophages mediate inflammatory damage through the Myd88/NFκB/NLRP3 pathway. (A) Pathway Enrichment analysis of Cluster 8 based on the GO database. (B) Images of the individual antibody staining for S100a9 and Myd88 in Figure 3E. n = 5. Scale bar, 50 μm.(C) Images of the individual Tunel staining in Figure 3L. n = 5. Scale bar, 50 μm.

Figure S5

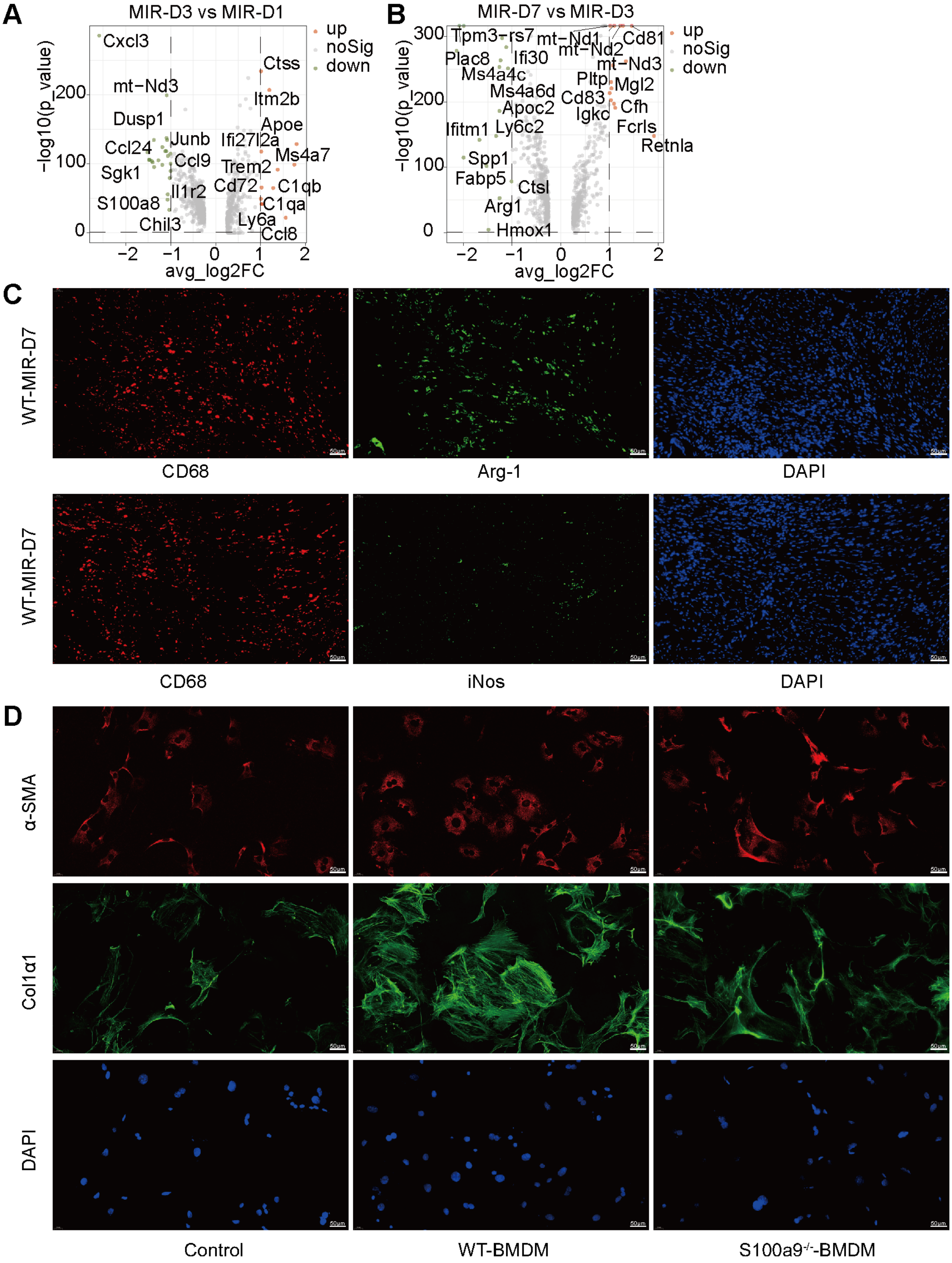

Figure S5: After MIR, macrophages gradually transition to a reparative M2 phenotype, promoting fibrotic remodeling. (A-B) Volcano plot of the fold-change in differential gene expression in day 3 post-MIR and Sham (left), day 7 post-MIR and day 3 post-MIR (middle) from all cells in the monocytes and monocyte-derived infiltrated macrophages. P-values were calculated by Wilcoxon rank sum test and P adjusted values were corrected by Bonferroni analysis. (C) Images of the individual

antibody staining for iNos and CD68, as well as Arg-1 and CD68 in Figure 4G. n = 5. Scale bar, 50 μm. (D) Images of the individual antibody staining for Col1α1 and α-SMA in Figure 4J. n = 5. Scale bar, 50 μm.

Figure S6

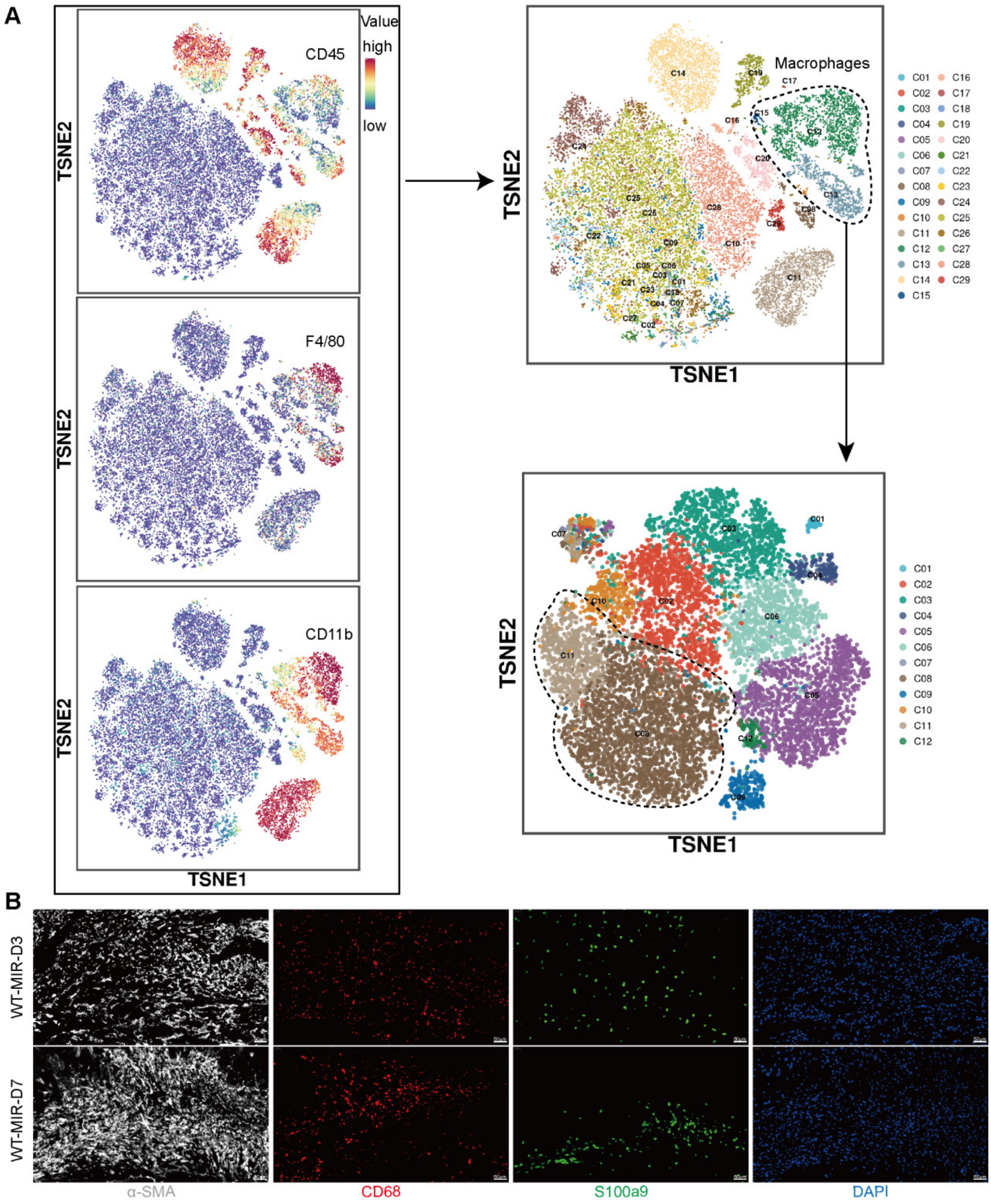

Figure S6: MMT in day 7 post-MIR . (A) Representative TSNE plots from CyTOF data show macrophage clusters after initial clustering of cells captured by mass cytometry. (B) Images of the individual antibody staining for α-SMA, CD68, and S100a9 in Figure 5B. n = 5. Scale bar, 50 μm.

Figure S7

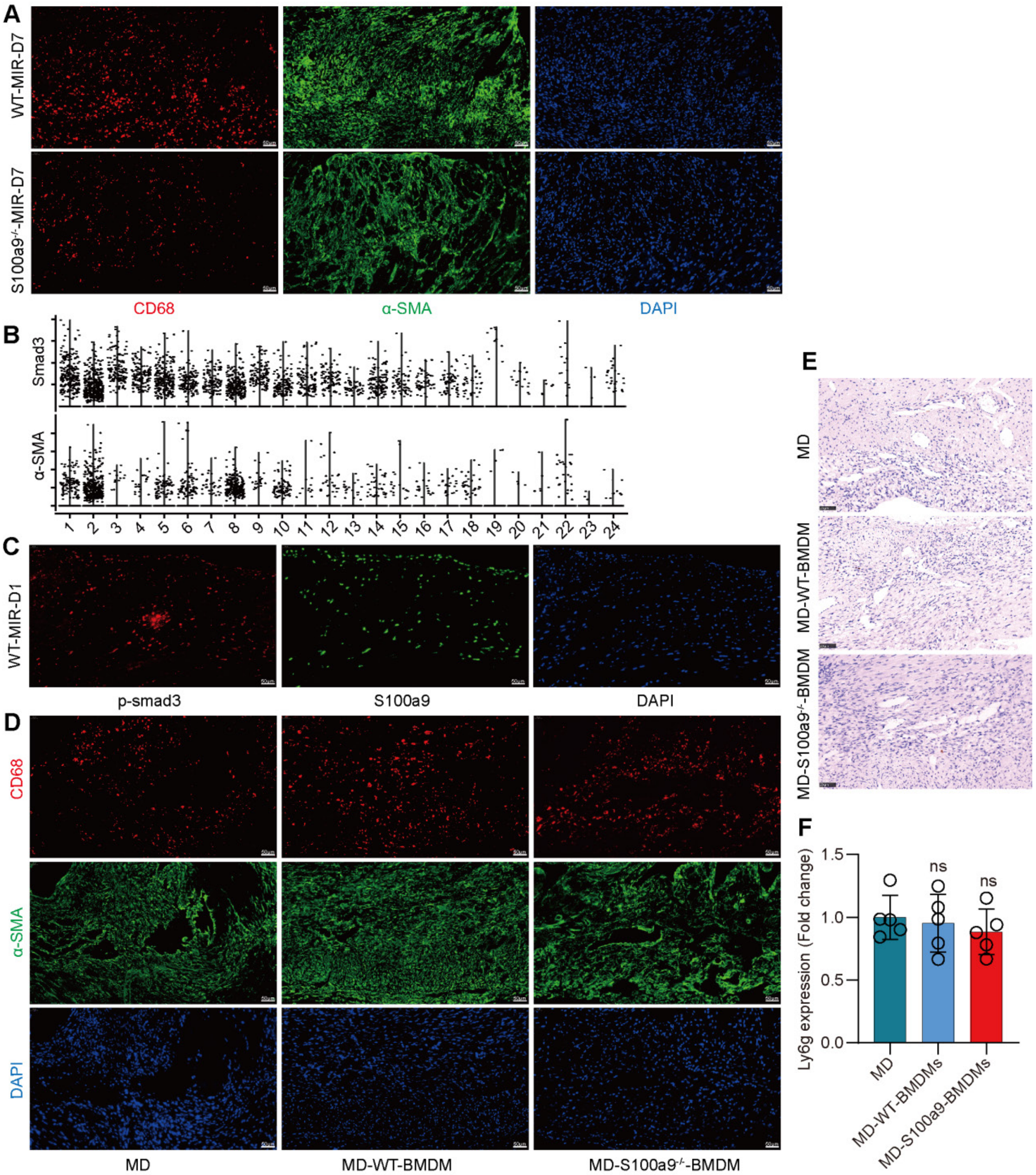

Figure S7: S100a9^{hi} macrophages highly express psmad3/α-SMA and other MMT-related genes. (A) Images of the individual antibody staining for CD68 and α-SMA in Figure 5C. n = 5. Scale bar, 50 μm. (B) Stacked violin plot of Smad3 and α-SMA in each MPC cluster. (C) Images of the individual antibody staining for p-smad3 and S100a9 in Figure 5E. n = 5. Scale bar, 50 μm. (D) Images of the individual antibody staining for CD68 and α-SMA in Figure 5J. n = 5. Scale bar, 50 μm. (E-F) Hearts were harvested at each group and heart sections immunostained with Ly6g and Quantification of Ly6g-positive area. n = 5. Scale bars, 50 μm. ns, no significance; *, P<0.05; **, P<0.01 compared to MD, one-way ANOVA.

Figure S8

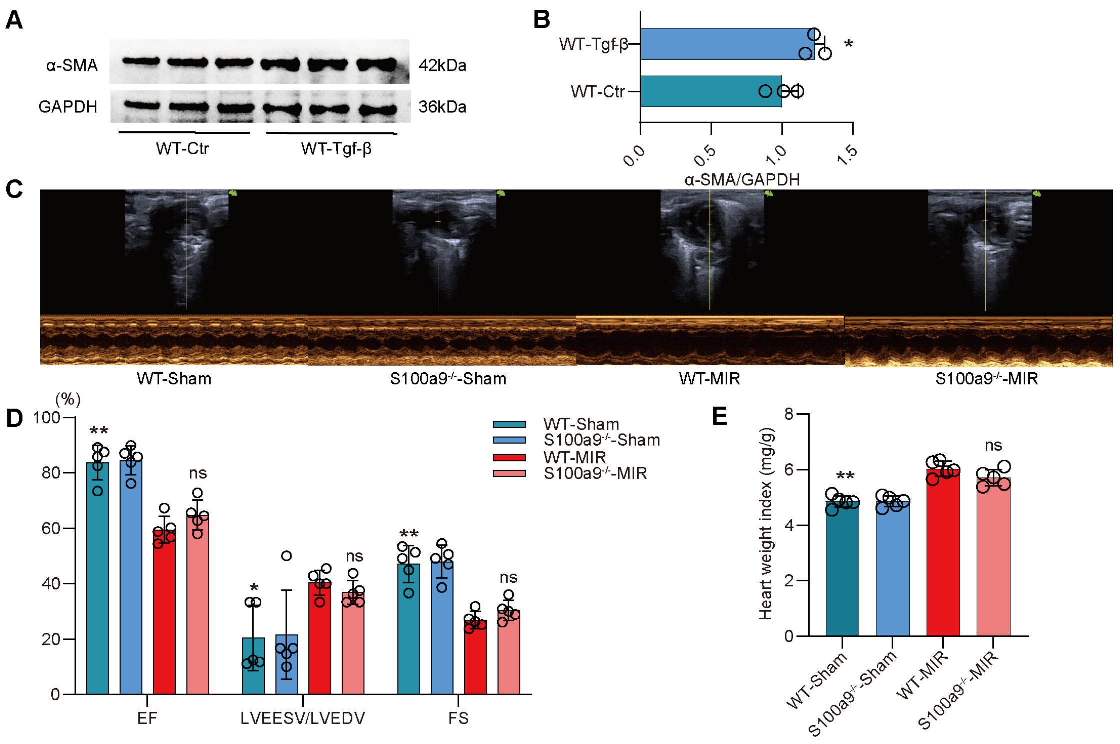

Figure S8: The effect of reinfusing s100a9$^{-/-}$ macrophages on myocardial fibrosis in mice after macrophage depletion and in vitro MMT validation. (A-B) Western blots of α-SMA and the quantification of expression in each group. n = 3 in each group. ns, no significance, * $P < 0.05$, ** $P < 0.01$ compared to WT-Ctr group, Student's t test. (C-D) Echocardiographic analysis of LV end-diastolic volume (LVEDV)/LV end-systolic volume (LVESV), and LV ejection fraction (EF) at day 30 after MIR or Sham surgery in WT and S100a9$^{-/-}$ mice. n = 5 in each group. ns, no significance, * $P < 0.05$, ** $P < 0.01$ compared to WT-MIR group, one-way ANOVA. (E) Heart weight index at day 30 after MIR or Sham group in WT and S100a9$^{-/-}$ mice. n = 5 in each group. ns, no significance, * $P < 0.05$, ** $P < 0.01$ compared to WT-MIR group, one-way ANOVA.

Figure S9

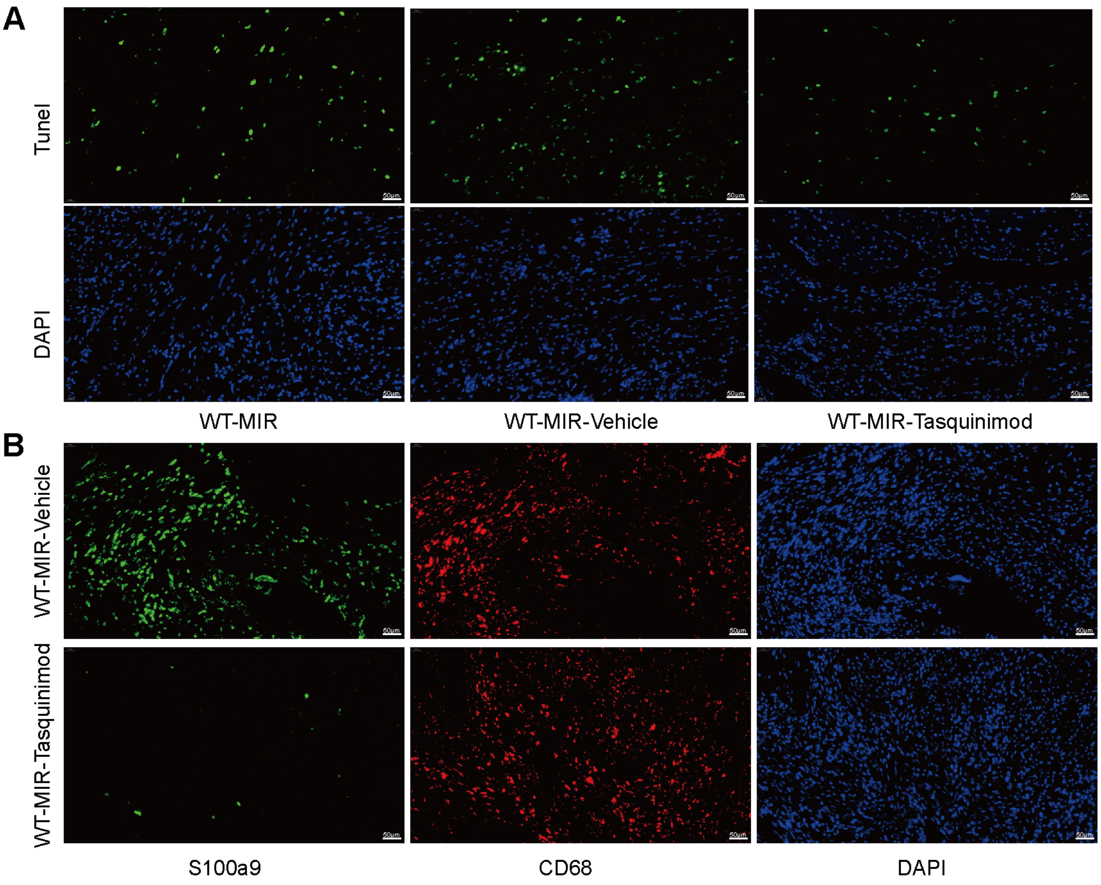

Figure S9: After pharmacological inhibition of s100a9, there is a reduction in the level of cell death and infiltration of s100a9^{hi} macrophages following MIR. (A) Images of the individual antibody staining for Tunel and DAPI in Figure 6G. n = 5. Scale bar, 50 μm. (B) Images of the individual antibody staining for CD68 and S100a9 in Figure 7A. n = 5. Scale bar, 50 μm.

Table S1. A total of 38,497 cells passed quality control and were classified into 26 clusters by scRNA-seq.

Table S2. A total of 17,393 cells were identified as MPCs and further divided into 25 clusters.
